# Supplementary material for: Thermoelectric current in a graphene Cooper pair splitter
Source: Nat Commun. 2021 Jan 8;12:138. doi: 10.1038/s41467-020-20476-7 (PMC7794233; doi:10.1038/s41467-020-20476-7)
Supplement: Supplementary file 1 — Supplementary Information [file 41467_2020_20476_MOESM1_ESM.pdf]

Supplementary Information: Thermoelectric current in a graphene Cooper pair splitter

Z. B. Tan,[1,2] A. Laitinen,[1] N. S. Kirsanov,[3,4,1,5] A. Galda,[6,7] V. M. Vinokur,[7,5]
M. Haque,[1] A. Savin,[1] D. S. Golubev,[8] G. B. Lesovik,[3,4] and P. J. Hakonen[1,8,*]

[1]*Low Temperature Laboratory, Department of Applied Physics, Aalto University, Espoo, Finland*
[2]*Shenzhen Institute for Quantum Science and Engineering, and Department of Physics, Southern University of Science and Technology, Shenzhen 518055, China*
[3]*Terra Quantum AG, St. Gallerstrasse 16A, 9400 Rorschach, Switzerland*
[4]*Moscow Institute of Physics and Technology, 141700, Institutskii Per. 9, Dolgoprudny, Moscow Distr., Russian Federation*
[5]*Consortium for Advanced Science and Engineering (CASE), University of Chicago, 5801 S Ellis Ave, Chicago, IL 60637, USA*
[6]*James Franck Institute, University of Chicago, Chicago, IL 60637, USA*
[7]*Materials Science Division, Argonne National Laboratory, 9700 S. Cass Ave., Argonne, IL 60439, USA*
[8]*QTF Centre of Excellence, Department of Applied Physics, Aalto University, FI-00076 Aalto, Finland*
(Dated: November 24, 2020)

This supplement provides additional details on experimental and theoretical analysis of our results. We begin with thermal modelling of our Cooper pair splitter device and discuss thermal transport in various parts of the sample, starting from the graphene heater and its electron-phonon coupling, down to small scale thermal gradients between the two quantum dots on SiO_2 (Supplementary Note 1). In Supplementary Note 2, we provide differential resistance data on the SGS superconducting junction thermometers and discuss how we can determine the thermal gradients in our samples using calibrations based on the SGS junctions. Additional thermopower data at finite bias is provided in Supplementary Note 3 in order to stress the importance of having a galvanically separated heater for inducing well-defined thermoelectric currents. Supplementary Note 4 contains details of our theoretical models. We present the theory of thermal transport in a system consisting of two quantum dots coupled to two separate normal leads and one common superconducting lead (cf. Supplementary Fig. 7 for the setting). In Supplementary Note 4 we describe the coherent transport model, which appears to agree well with the experimental results. In Supplementary Note 5, we discuss the incoherent transport regime. This model helps to account for additional sign changes in the local thermoelectric current at low heating power which are seen in the experiments, but not captured by the coherent model.

SUPPLEMENTARY NOTE 1: MODELING HEAT TRANSPORT IN THE SYSTEM

Supplementary Fig. 1 displays a schematic heat flow diagram of our graphene Cooper pair splitter device. The heater, the graphene Josephson junctions thermometers (SGS_L and SGS_R), and the quantum dots (QD_L and QD_R) are all lying on a SiO_2/Si substrate. We have left out the Al electrode which connects thermally QD_L and QD_R owing to quasiparticle resistance. According to theory, the important temperatures for non-local thermoelectric effects are those of the left and right normal reservoirs, T_L and T_R which are formed by the large graphene pieces before the narrow graphene constrictions making the connections to the dots. For the local thermopower also the temperature of the superconductor, T_S, is relevant.

Thermal gradient between the two dots is produced by heat flow imposed across the substrate underneath the sample. Establishment of local substrate temperature requires sufficient amount of scattering of phonons, i.e. small enough mean free path for them (see Sect. D 1). In our analysis we assume that the local temperatures T_L, T_S, and T_R can be approximately defined on sub-micron length scales, which is on the order of the separation of the two graphene reservoirs with the SGS thermometers.

The thermal analysis of our system can be divided into four parts: 1) thermal flow in the heater, 2) thermal flow from the heater to the substrate, 3) thermal flow on the SGS thermometer, and 4) thermal flow along the substrate.

A. Thermal flow in graphene heater

The simplest model for the temperature rise in the graphene ribbon heater is provided by the hot electron model. This allows us to estimate the electronic temperature of the graphene electrons using the formula: $T_e = \frac{\sqrt{3}}{8}\frac{eV}{k_B}$, which yields $T_e^{\max} \sim 80$ K at the highest employed heating voltages. This temperature represents an average over the heater electrons and the actual

* Corresponding author: pertti.hakonen@aalto.fi

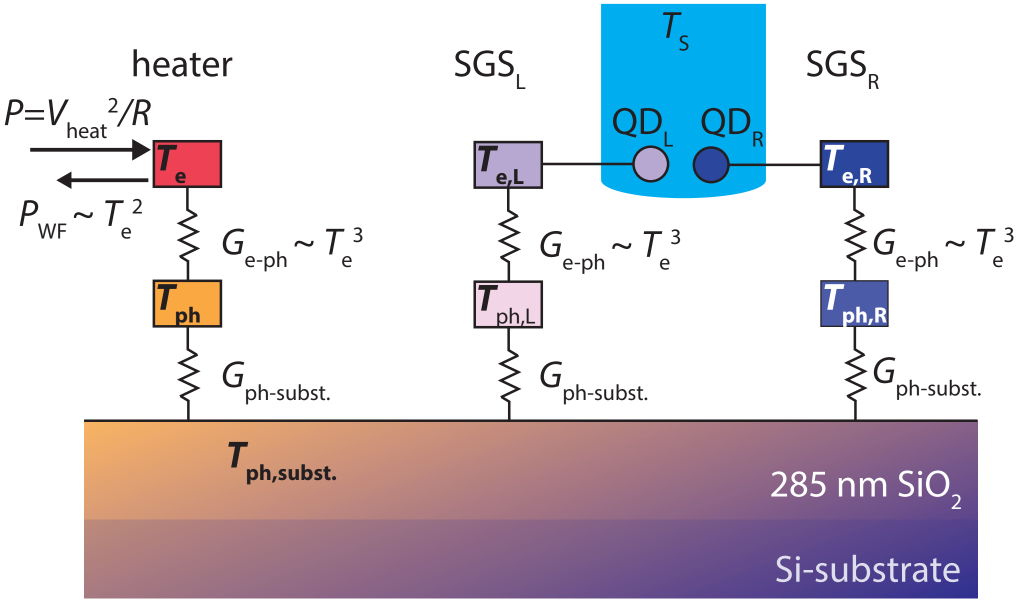

Supplementary Fig. 1. **Heat flow model across the 8-μm-long sample.** The scheme displays electron and phonon systems in graphene (heater, and superconducting junctions SGS_L and SGS_R), as well as phonon system of the substrate. Heat is brought in by Joule heating $P = V_h^2/R_h$ with $R_h \sim 20$ kΩ at a typical charge carrier density of $n = 4 \times 10^{11}$ cm^{-2}. The electron-phonon coupling in the graphene ribbon heater is expected to be dominated by impurity-assited acoustic phonon scattering (supercollisions) [1], while the phonon-phonon heat transport to the substrate is weakened by Kapitza mismatch at the interfaces. Compared with thermal conductivity of the strongly doped Si, the Kapitza resistance between Si and SiO_2 can be neglected (The Kapitza resistance corresponds to approximately 4 μm layer of our doped Si.) For electrically conducting parts, the electrical heat diffusion governed by the Wiedemann-Franz law is included as relevant. Warm (reddish) colors indicate higher temperatures (close to $T_{e,max} = 80$ K), while bluish colors denote lower temperatures (close to the base temperature $T = 90$ mK). The total thickness of the sample is 525 microns (not to scale).

temperature distribution over the wire is approximately quadratic with the end temperatures fixed according to the heat balance in the graphene reservoirs of the ribbon.

In order to evaluate the validity of the hot electron model, let us consider the behavior of electronic temperature at the ends of a wire coupled to two wide graphene leads as depicted in Supplementary Fig. 2.

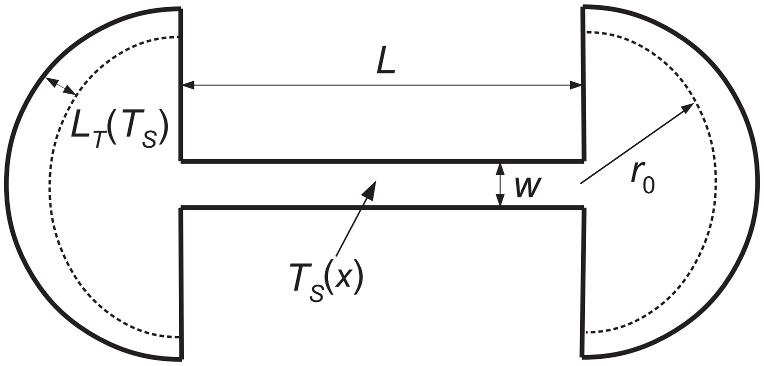

Supplementary Fig. 2. **Model geometry of the graphene ribbon heater.** The heater (size $L \times w$) is connected to two wide, half-infinite-plane graphene reservoirs at the ends. This geometry was employed for the analysis of the relaxation of T_e outside the heater section. The radius r_0 determines the border of the region where electronic heat diffusion can be regarded as small.

The temperature both in the ribbon and in the graphene leads can be found from the basic heat transport equation

$$\frac{\pi^2 k_B^2 \sigma}{6e^2} \nabla^2 T_e^2 + \frac{j^2}{\sigma} - \Sigma_{sup}(T_e^3 - T_{ph}^3) = 0, \tag{1}$$

where σ is the conductivity per square, j is the current per unit width ($j = I/w$) and the electron-phonon heat flow per unit area

equals $\Sigma_{sup} = 7.5\times10^{-4}\frac{D^2\tilde{n}_s}{k_F\ell}$ according to the experimental supercollision results [1, 2]; here $D \simeq 70$ eV denotes the deformation potential (in eV) and $\tilde{n}_s$ is the charge carrier density (in units of 10^{12} cm^{-2}). Using the above numbers, we obtain approximately equal heat fluxes for electron-phonon coupling in the heater and the electronic heat flux at the heater ends. Thus, $T_e^{\max}$ becomes lowered to ~ 50 K.

Outside the narrow section of the heater, $j = \frac{I}{\pi r}$ in the graphene lead at a distance r from the end of the ribbon. At large bias, electron-phonon coupling dominates and the temperature in the graphene near the heater ends equals to

$$T_S(r) = \left(T_{ph}^3 + \frac{j(r)^2}{\sigma\Sigma_{sup}}\right)^{1/3}. \tag{2}$$

A characteristic length scale for the decay of the heat flow along graphene reservoir can be obtained by equating the local variation of the gradient term in Supplementary Equation 1 to the change in heat flux due to electron-phonon coupling. The characteristic thermal relaxation length scale $L_T(T_S)$ becomes

$$L_T(T_S) = \sqrt{\frac{\pi^2 k_B^2 \sigma}{6e^2\Sigma_{sup}T_S}}. \tag{3}$$

At $T_S = 1$ K, we obtain $L_T = 2.3$ μm using a carrier concentration of $n_s = 4\times10^{11}$ cm^{-2} which is close to our experiments at a back gate voltage 5 V. Since this length decreases with temperature, it is evident that T_e decreases quickly in the leads and that most of the heating power is deposited to the substrate within and near the heater.

The Kapitza resistance between SiO_2 and graphene will enhance T of graphene phonons upto ~ 2 K, when the electronic temperature is 10 K. This will change slightly the above relaxation length, but it is still the supercollision cooling with coupling $\Sigma_{sup}(T_e^3 - T_{ph}^3)$ that governs thermal relaxation of the electrons.

B. Thermal flow from the heater to the substrate

On the basis of the smallness of the thermal relaxation length in Supplementary Equation 3, we assume that most of the heater power is deposited to the substrate within the area of the graphene ribbon heater and its immediate neighborhood. Using 2 μm^2 for the thermal contact area, $A_K = 500$ Wm^{-2}K^{-4} for Kapitza conductance ($P = \frac{1}{4}A_K(T_h^4 - T_s^4)$ with temperatures T_h and T_s on the opposite sides of the interface) [3], we obtain a phonon temperature of $T_h \sim 9$ K in graphene at 40 mV heating voltage. This elevated phonon temperature will relax along the substrate via phononic thermal conductance.

C. Thermal flow on the SGS thermometer

The SGS thermometers are formed by two-terminal, Al/Ti-contacted graphene parts in the center of large pieces of graphene. They are made in such a way that there is an uninterrupted path of non-superconducting graphene connecting the thermometer to a quantum dot. This guarantees smooth thermal flow along the thermometer area without any strong suppression in the heat conductivity due to proximity-induced superconducting gap (note that the graphene electrode is fully crossed by a superconducting lead at the further end of the thermometer, see Fig. 1 of the main paper). The purpose of these SGS devices is to track the temperature of the electronic graphene reservoirs that govern the distribution of incoming electrons/holes on the graphene quantum dots. The large area of the SGS devices guarantees that their temperature will well track the temperature of the substrate, independent of a possible heat input coming along the graphene ribbons from the dots.

The operating range of our SGS thermometers is up to 0.8 K, which facilitates thermopower studies up to rather substantial thermal gradients. At a typical thermometer temperature of 0.5 K, we obtain a characteristic relaxation length of 3.3 μm using Supplementary Equation 3. This is larger than the extent of thermometer along the heat flow direction. Consequently, we will take a spatially independent temperature for the SGS thermometer. In order to determine how the SGS thermometer averages over the spatially dependent substrate temperature $T_s(x)$, we assume that the heat flow balance between SGS and the substrate remains zero. This yields the integral condition $\int(T_{SGS}^3 - T_s^3(x))dx = 0$. At low temperature with small $dT_s^3(x)/dx$, one may obtain a simple estimate for $T_{SGS} \simeq \left[\frac{1}{2}(T_l^3 + T_r^3)\right]^{1/3}$ for the temperature recorded by the thermometer; here T_l and T_r denote the temperature at the left and right edges of the thermometer region, respectively.

D. Thermal flow along the substrate

1. Local phonon temperature and heat diffusion

For application of the regular heat diffusion equation, small mean free path of phonons is essential. In clean silicon, the mean free path of phonons can be on the order of 100 microns, which would lead to problems in defining a proper substrate temperature across our CPS device having a size smaller than 10 μm. However, the interfacial scattering at the SiO_2/Si interface and the strong impurity doping of the Si^{++} material decrease the mean free path of phonons substantially. Typically in case of thin layers and rough surfaces at an interface, the phonon mean free path is limited to a value of the order of layer thickness [4] (Casimir limit). Thus, in our case $\ell_{ph} \sim 300$ nm in the SiO_2 that is in thermal contact with the CPS device. Owing to the relatively small ℓ_{ph}, we argue that the heat diffusion equation can be employed to deduce approximate temperature distribution along the substrate across the area of the graphene reservoirs of our Cooper pair splitter.

2. COMSOL simulations for temperature difference between the dots

The heat diffusion equation was solved using COMSOL multiphysics. The geometrical model used in the simulations (see Supplementary Fig. 3) is a simplified version of the real device presented in Fig. 1 of the main text. Our simulations using exact sample dimensions focused on the temperature profile on the surface along the line drawn through the heater and both quantum dots. Best available data on materials parameters were employed in calculating thermal gradients along the substrate. The graphene heater power was inputted only over the actual heater size, which slightly increased the maximum temperature of the SiO_2 but this was deemed irrelevant further away from the heater which was the main region of interest.

In order to achieve most realistic temperature profile, we employed temperature dependent heat conductivities both for the phonon thermal conductance as well as for the metallic heat diffusion. The mean free path of phonons influences strongly the thermal conductivity (see Sect. D 1). We employed $\kappa_{SiO_2} = 0.01 \times T^2$ W/(mK^3) [5], $\kappa_{gr} = 0.02 \times T^2$ W/(mK^3) [6, 7], and $\kappa_{Si^{++}} = 0.01(T/K)^2$ W/mK^3 [8] for thermal conductivities of SiO_2, graphene, and strongly doped silicon, respectively. For dynamical calculations, we also specified heat capacities $C_{SiO_2} = 3 \times 10^{-3} \times T^2$ J/(kgK^3), $C_{gr} = 1 \times T$ J/(kgK^2), and $C_{Si^{++}} = 10^{-3}$ J/(kgK^3), respectively.

Supplementary Fig. 3 displays simulation results for the spatially dependent temperature $T(x)$ on the surface of the SiO_2 layer as a function of the distance x from the heater along the central symmetry axis intersecting the two quantum dots and the center of the graphene ribbon heater. The simulated trace at $V_h = 40$ mV yields $\Delta T = 72$ mK for the temperature difference between the two red lines marking the edges of the SGS graphene thermometers. The flatter regions before and after the steep section between the red lines is due to the large phononic conductance of graphene. Note that the regions would be even flatter if the electron-phonon coupling and the electronic heat conductance κ_e in graphene would have been included. This omission of κ_e also renders the modification of electrical thermal conductivity by the induced superconducting gap irrelevant for the simulation.

The COMSOL simulation was also used to estimate the thermal time constant of the system. This was done by looking at the phase difference between the heater temperature T_h and T_{QD_L} as the heater modulation frequency was increased. The results are presented in Supplementary Fig. 3d at frequencies 0.1, 1, and 2 MHz. The simulation demonstrates that at 100 kHz the phase difference is still negligible, while at higher frequencies it starts to grow. Thus, we can conclude that the time constant $\tau < 0.01$ ms and steady state approximations can safely be applied to our measurements at $f < 10$ Hz.

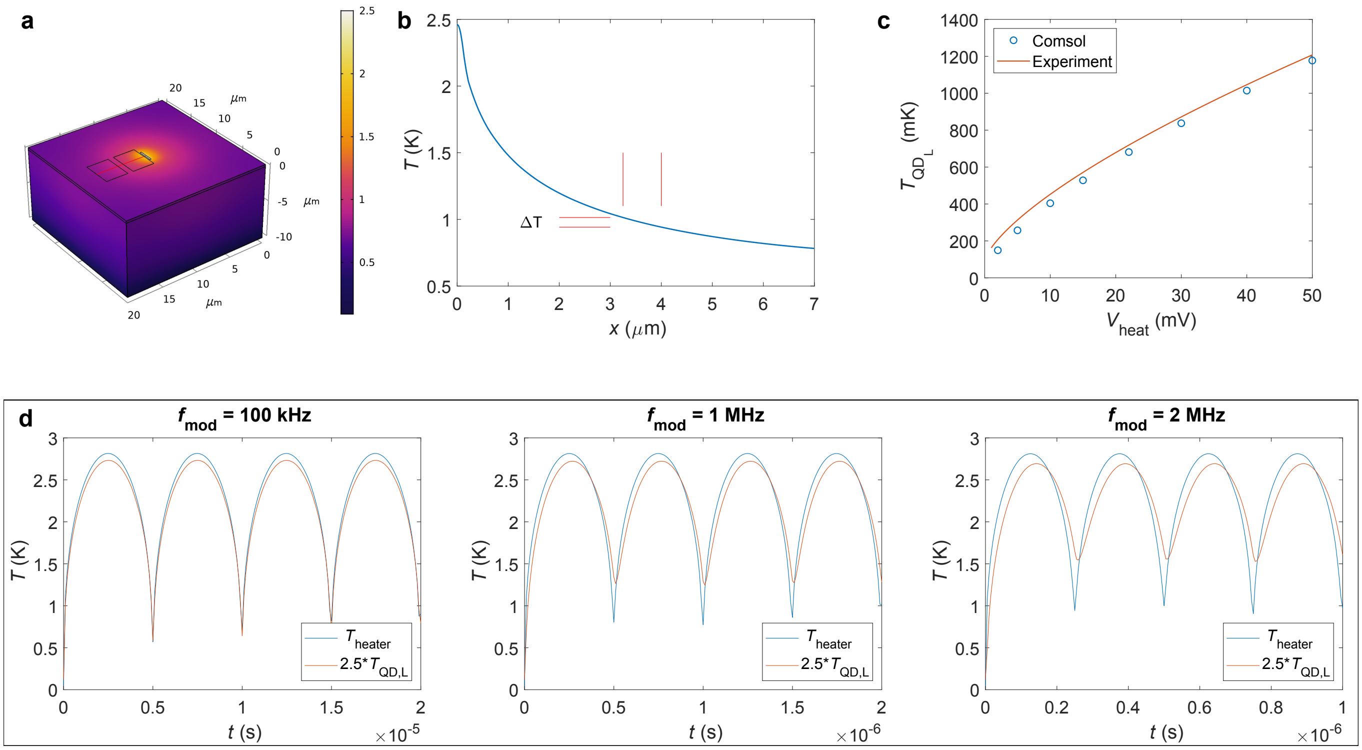

Supplementary Fig. 3. **Simulation results.** **a** 3D model used in the COMSOL simulations. The large block represents the 525 μm thick doped Si substrate, the layer on top of that is 300 nm of SiO_2, and the three rectangles on top of SiO_2 represent graphene pieces on top of the SiO_2 layer. Brighter (yellow) color indicates higher temperature. **b** Simulated temperature at V_h = 40 mV depicted along the red line in **a** on the left, from the center of the heater through the quantum dots. The red markings indicate the locations of graphene reservoir regions (the edges of the area where the SGS thermometers are located) and the corresponding temperature drop $\Delta T \simeq 72$ mK. **c** Temperature at the left quantum dot obtained from the Comsol model (blue dots), and experimental heater calibration of Supplementary Equation 4 plotted as a function of the heater voltage. **d** Temperature at the heater (blue) and $T_{QD,L}$ (red) presented as a function of time when the heater power is modulated sinusoidally at maximum heating power (V_h = 40 mV) using frequencies f = 0.1 MHz, 1 MHz, and 2 MHz indicated on top of the figures.

SUPPLEMENTARY NOTE 2: CALIBRATION OF HEATER VOLTAGE VS. SGS JUNCTION TEMPERATURE

Once calibrated, the heater voltage could be used for specifying the temperature difference across our Cooper pair splitter. This was useful, in particular because of the poor resolution of the SGS_R thermometer, which imposed long measurement times for good accuracy. The poor resolution of SGS_R was due to lack of measurement wires in the cryostat, which forced us to connect the SGS_R thermometer in a 2-wire configuration.

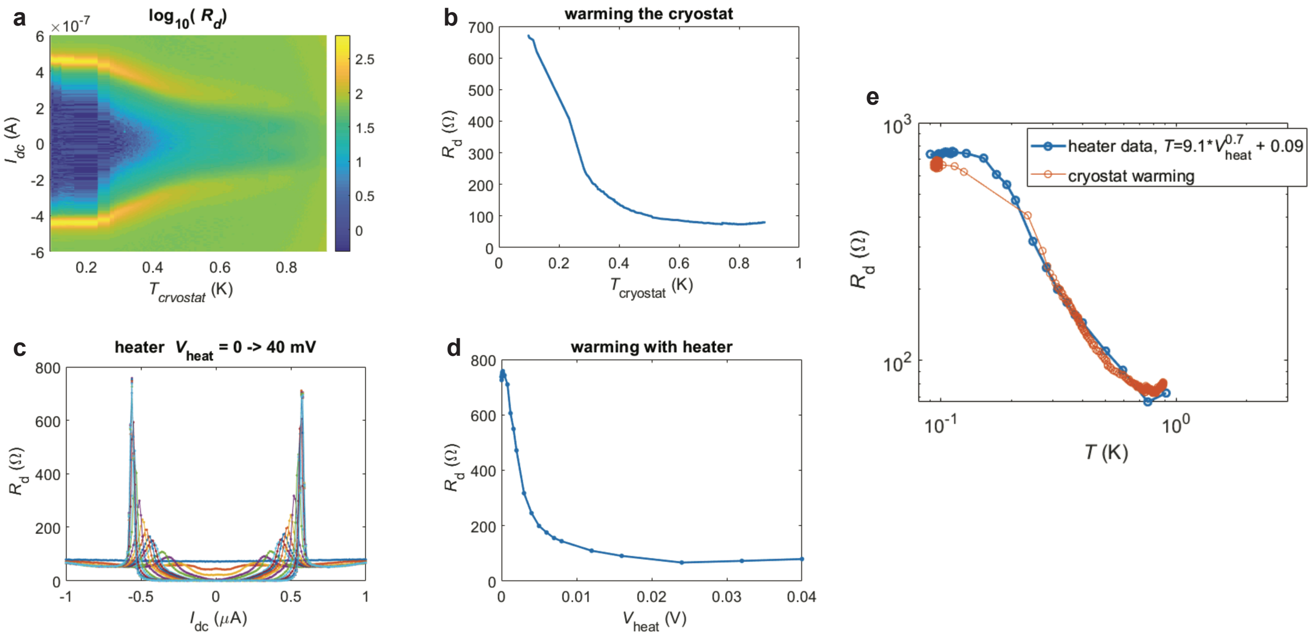

Supplementary Fig. 4. **Measurement data sets employed for obtaining a relation between the heater voltage and the SGS junction temperature.** **a** Logarithm of the differential resistance of the SGS_L junction displayed on the cryostat temperature vs. dc bias current plane. **b** Peak resistance value of R_d vs. equilibrium temperature $T_{cryostat}$ while warming the cryostat. **c** Differential resistance of the SGS_L junction vs. dc bias current at different heating powers applied to the galvanically-separated graphene ribbon heater ($V_h = 0 \ldots 40$ mV). **d** Peak resistance value $R_d^{peak} = \max R_d(I_{DC})$ vs. heating voltage. **e** Data for the peak value R_d^{peak} scaled to match the maximum sub-gap value of $R_d(T)$ using Supplementary Equation 4.

The temperature of SGS_L *w.r.t.* the heater voltage was calibrated by measuring the IV characteristics of the SGS_L as a function of the heater voltage V_h and the equilibrium cryostat temperature $T_{cryostat}$. The obtained differential resistance R_d characteristics are presented in Supplementary Fig. 4, in which the decrease of the superconducting gap with increasing V_h and $T_{cryostat}$ can be seen clearly. At the edge of the gap, we traced the maximum differential resistance, $R_d^{peak} = \max R_d(I_{DC})$, both as a function of V_h and T_{cryo}. The observed traces can be made to overlap each other by adjusting the x-scale, i.e. by mapping the voltage scale to a corresponding temperature scale. The obtained scaling relation for SGS_L is given by:

$$T_L = (9.1 \cdot V_h^{0.7} + 0.09)\ \text{K}, \tag{4}$$

where V_h is in volts. Note that there is an offset of 90 mK that we interpret as the effective electron temperature in graphene at the cryostat base temperature when no heating is applied. For SGS_R, we obtain the relation $T_R = (8.4 \cdot V_h^{0.7} + 0.09)$ K .

Note that the exponent of the heater voltage, 0.7, is approximately consistent with $T_L^3 \propto V_h^2$ (and $T_R^3 \propto V_h^2$) that would in turn be consistent with the cooling $P \propto T^3$ being dominated by acoustic phonons, for which the thermal conductivity $\kappa_{ph} \propto T^3$.

As mentioned in the Methods section, the length of our SGS thermometers is intermediate between the short and long junction limits. Unfortunately, no analytic formulas exist for this regime. For modeling purposes, we have employed the low temperature expression for the critical current of long junctions $I_c = E_{th} b(1{-}1.3 \exp{[-bE_{th}/3.2k_bT]})/eR_n$, using b as a fitting parameter; here R_n is the normal state resistance and k_b denotes Boltzmann's constant. Using $b \simeq 2.2$, this formula agrees approximately with the measured behavior of $I_c(T)$ and the I_cR_n product at the lowest temperatures. The fit also corroborates with the observed optimum thermometer sensitivity around $T \simeq 0.3$ K, with tendency towards saturation below $T < 0.2$ K. However, the thermometer works even below 0.2 K as the maximum differential resistance in the sub gap region still grows with decreasing temperature below $T = 0.2$ K. The absence of clear hysteresis of the SGS junctions is assigned to the built-in shunts provided by the surrounding, non-proximitized graphene.

The calibration between heating-induced temperature and cryostat temperature is bound to the site where the SGS junctions are located. It is true that our thermometers are some distance apart from the quantum dots. Thus, there may be small thermal gradients along the graphene part in contact with the thermometer. The gradient depends on the ratio of electron-phonon/Kapitza conductance to the substrate (as discussed in relation to Supplementary Fig. 1) and the electronic conductance along graphene. At the lowest temperatures, the electronic conductance will dominate and we may neglect the thermal gradients along the graphene flake. At higher temperatures, thermal gradients will appear and the measured temperature difference will be an upper bound for the temperature difference. However, if we plot the local thermoelectric data for the peak 4 in Fig. 4a of the main text as a function of temperature difference $\Delta T = T_L - T_S$, we find a linear dependence with ΔT up to the largest heating powers employed in the experiment, as demonstrated by the data in Supplementary Fig. 5. We consider this as evidence that thermal gradients are not disturbing our calibration significantly at those temperatures relevant for our analysis.

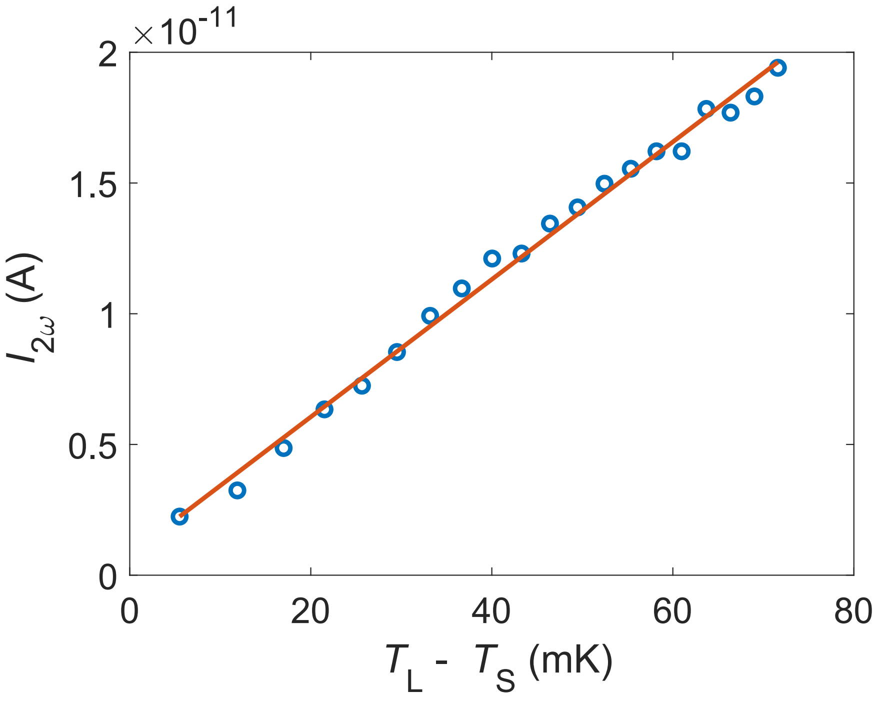

Supplementary Fig. 5. **Local thermoelectric current in the left quantum dot as a function of heating-induced temperature difference**. The temperature difference $T_L - T_S = (T_L - T_R)/2$ is determined using SGS junctions, calibrated against the cryostat temperature. The data are measured from the peak no. 4 in Fig. 4 of the main text. The measured points display linear dependence on $T_L - T_S$, which extends all the way up to the largest heating powers.

SUPPLEMENTARY NOTE 3: ADDITIONAL DATA ON BIAS-INDUCED THERMOPOWER

The thermoelectric current measured in the main paper was purely driven by temperature gradient, without any bias applied on the Cooper pair splitter. We also performed experiments where the thermoelectric response was investigated without a separate heater. Bias current in the graphene ribbon connecting the QDs to the graphene reservoirs leads to heating which creates basically local thermopower phenomena with symmetry properties close to the non-local phenomena. Because of this, the central figures in the main paper (Figs. 3 and 4) contain only data with plain thermal drive.

Supplementary Fig. 6 shows results on thermoelectric current in a Cooper pair splitter driven by a voltage bias on one of the quantum dots. In this case, thermoelectric effects and the bias-induced CPS and EC will mix together in the behavior. Supplementary Fig. 6a and 6b display the currents of the two dots I_L and I_R measured at ω as function of the dc bias V_b and the side gate voltage $V_{sg,L}$ on QD_L; here dc and ac voltage bias is applied only on QD_R with the middle superconcucting lead grounded. The current I_L shows different sign with positive and negative dc bias V_b on QD_R. In addition, I_L changes its sign when $V_{sg,L}$ crosses $V_{sg,L} = 0.72$ V, at which the energy level of QD_L matches the Fermi energy. Ac current I_R, however, is insensitive to the $V_{sg,L}$.

Figs. 6c and 6d display I_L and I_R as functions of dc bias V_b on QD_R and the side gate voltage $V_{sg,R}$. Here I_L changes its sign, when V_b crosses zero, but it retains its sign when $V_{sg,R}$ crosses $V_{sg,R} = 0.535$ V, at which the energy level of QD_R matches the Fermi level. Note that the observed variation of I_L as function of $V_{sg,L}$, $V_{sg,R}$ and V_b can be explained by considering heating due to V_b and local thermoelectric currents induced by the ensuing thermal gradients between QD_L and the superconductor. Unfortunately, there are no means to single out the non-local thermoelectric effects in these sets of data.

If QD_R is ac biased at ω without any dc, we obtain Supplementary Fig. 6e for the current $\tilde{I}_L$ in QD_L at 2ω. The thermoelectric current in QD_L changes its sign while the energy level tuned by $V_{sg,L}$ crosses the Fermi energy. On the contrary, $\tilde{I}_L$ keeps its sign as the energy level is tuned by $V_{sg,R}$ across the Fermi surface. These results are consistent with those in Figs. 6a-d.

Although the observed data in Supplementary Fig. 6 are similar to those in Figs. 3 and 4 in the main paper, and apparently non-local effects according to this similarity in symmetry and asymmetry, they can be explained using bias-induced heating and local thermoelectric effects induced by thermal gradients. In Figs. 6a and 6c, the dc and ac bias applied to QD_R causes heating in the middle Al lead. When the dc bias is positive, the heating in the Al lead will be in the same phase with the ac bias of QD_R: i.e. the heating in Al follows the maximum and minimum of the ac bias on QD_R. When the dc bias is negative, however, the maximum heating will coincide with minimum of the ac, and the heating in the middle Al will be phase shifted by π from the ac bias on QD_R. Hence, in Figs. 6a and 6c, the thermoelectric current I_L has an opposite sign at positive or negative dc bias. In Supplementary Fig. 6a, I_L changes its sign due to the same reason as in Fig. 2a of the main text when $V_{sg,L}$ crosses $V_{sg,L} = 0.675$ V. Because the heating in the middle Al lead is equal at left and right fo the conductance peak of QD_R, I_L keeps the same sign while energy level of QD_R crosses Fermi level in Supplementary Fig. 6c.

Even when using the 2ω method, driving QD_R at ω, measuring I_L at 2ω, the same heating argument can be employed to explain the observed bahavior in Supplementary Fig. 6e. In the absence of bias voltage, the heating is now at 2ω, at which the signal then appears. These data have strong resemblance with the result in Fig. 4d in the main paper. The current I_R at ω is depicted in Supplementary Fig. 6f.

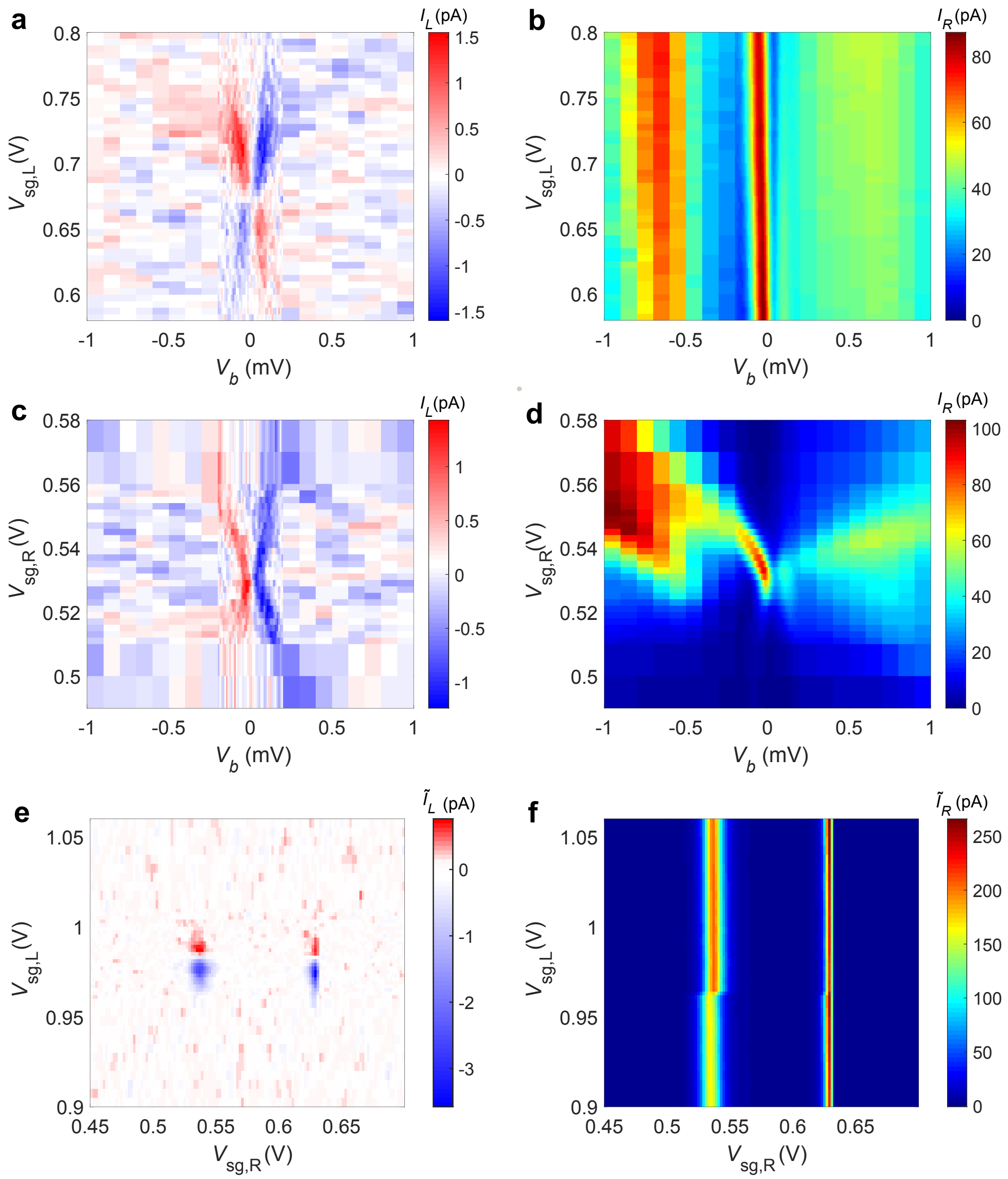

Supplementary Fig. 6. **Splitting at finite bias: competition of CPS, EC and local thermoelectric effects.** **a** Ac current measured on QD_L as a function $V_{sg,L}$ while sweeping the dc bias V_b on QD_R at $V_{sg,R}$ = 0.535 V. **b** Ac current of QD_R as a function of $V_{sg,L}$ while sweeping dc bias on QD_R at $V_{sg,R}$ = 0.535 V. **c** Ac current of QD_L as a function of $V_{sg,R}$ while sweeping dc bias on QD_R at $V_{sg,L}$ = 0.72 V. **d** Ac current of QD_R as a function of $V_{sg,R}$ while sweeping dc bias on QD_R at $V_{sg,L}$ = 0.72 V. In all of these measurements a-d, QD_R was also biased using 20 μV_{rms} at 2.1 Hz for ac measurements. **e** Apparent non-local thermoelectric effects due to variation in Joule heating across the resonance. Current $\tilde{I}_L$ (at 2ω) of QD_L measured over $V_{sg,L}$ and $V_{sg,R}$ plane using an ac bias of 50 μV on QD_R. **f** The corresponding current I_R of QD_R measured at ω over $V_{sg,L}$ and $V_{sg,R}$ plane. Notice the resemblance of frame **e** with the non-local thermoelectric current patterns in Fig. 3 of the main paper. Here, the large ac excitation leads to gate voltage dependent heating in QD_R, which leads to variation of T with the same symmetry properties as the non-local thermoelectric effect with constant thermal gradient.

SUPPLEMENTARY NOTE 4: THEORETICAL MODELING: COHERENT TRANSPORT IN N-DOT-S-DOT-N SYSTEM

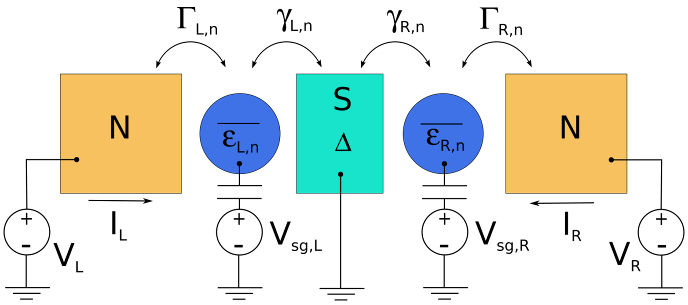

Supplementary Fig. 7. **Schematics of the N-dot-S-dot-N system.** Two quantum dots with the energy levels $\varepsilon_{L,n}$ and $\varepsilon_{R,n}$ are coupled to the common superconducting lead with the gap Δ and the temperature T_S, and to the two different normal bulk leads with the temperatures T_L (left lead) and T_R (right lead). The tunneling rates between the dots and the superconductor are $\gamma_{L,n}$ and $\gamma_{R,n}$, while the tunneling rates between the dots and the normal leads are $\Gamma_{L,n}$ and $\Gamma_{R,n}$. Bias volatges V_L and V_R can be applied to the outer leads, and the side gate potentials $V_{sg,L}$ and $V_{sg,R}$ allow one to shift the energy leves $\varepsilon_{L,n}$ and $\varepsilon_{R,n}$. The potential of the superconductor is supposed to be zero. The electric currents I_L and I_R flow through the quantum dots into the superconductor.

In this note we outline the theory of thermal transport in the system consisting of two quantum dots coupled to two separate normal leads and one common superconducting lead. The schematics of it is shown in Supplementary Fig. 7. Here, we describe the coherent transport regime, which rather well describes the main findings of our experiment. The additional sign changes in the local thermoelectric current at low heating power which are not captured by the present description are explained within the incoherent model discussed in Supplementary Note 5.

We assume that the conductances of the dots are high enough so that the dots enter the Fabry-Pérot regime, and one can ignore Coulomb blockade. We split the energy levels in both dots into two groups: well coupled to the leads levels $\varepsilon_{j,n}$ (here the index $j = L, R$ indicates the left and right dot, respectively, and n enumerates the energy levels within one dot), and poorly coupled to the leads levels $\varepsilon'_{j,n}$. The hopping rates of electrons between the levels $\varepsilon_{j,n}$ and the normal leads, which in our experiment are big graphene flakes, are denoted by $\Gamma_{j,n}$, while the hopping between the dots and the superconducting lead is set by rates $\gamma_{j,n}$. The energy levels can be tuned by side gate potentials $V_{sg,j}$ as

$$\varepsilon_{j,n}(V_{sg,j}) = a_j(V_{sg,j} - V_{\max,j,n}), \quad \varepsilon'_{j,n}(V_{gj}) = a_j(V_{sg,j} - V'_{j,n}). \tag{5}$$

Here the constants a_j are determined by the ratios of the gate and the dot capacitances, and $V_{\max,j,n}$ are the gate voltages at which the conductance of the corresponding dot exhibits a peak and reaches a local maximum, and $V'_{j,n}$ are the gate voltages at which the energies of the dark states $\varepsilon'_{j,n}$ become equal to zero. From the experiment we approximately find

$$a_L \approx 0.003\ \frac{\text{eV}}{\text{V}}, \quad a_R \approx 0.01\ \frac{\text{eV}}{\text{V}}. \tag{6}$$

Bias voltages V_j are applied to the metallic leads, while the potential of the superconductor equals zero.

A. Local electric current in the normal state

In this section, we recall some well known results and consider fully normal system assuming $\Delta = 0$. For a nice general review on thermoelectric effects in normal three terminal systems we refer the reader to Ref. [9]. Since we neglect the Coulomb blockade, transport of charge through the quantum dots is well described by the Landauer formula,

$$I_j^{\text{loc}} = \frac{2e}{h}\int dE\, \tau_j(E, V_{sg,j})[f_j(E - eV_j, T_j) - f_S(E, T_S)], \tag{7}$$

where the superscript loc indicates the local character of the current (i.e. I_j^{loc} can be evaluated assuming that the other quantum dot does not exist), h is Planck's constant, $\tau_j(E, V_{sg,j})$ is the transmission probability of the dot,

$$f_j(E - eV_j, T_j) = \frac{1}{1 + e^{(E-eV_j)/k_B T_j}}, \quad f_S(E, T_S) = \frac{1}{1 + e^{E/k_B T_S}} \tag{8}$$

are the electron distribution functions in the bulk normal lead j and in the superconductor, V_j is the electric potential applied to the lead j, T_j is the temperature of the lead j, and T_S is the temperature of the central electrode. The factor 2 in front of

Supplementary Equation (7) accounts for the spin degeneracy. We use two models for the transmission probability: (i) simple resonant tunneling model, in which $\tau_j(E, V_{sg,j})$ is given by a sum of Lorentzian peaks, each coming from one of the energy levels,

$$\tau_j(E, V_{sg,j}) = \sum_n \frac{\gamma_{j,n}\Gamma_{j,n}}{\left(E - \varepsilon_{j,n}(V_{sg,j})\right)^2 + \frac{(\gamma_{j,n}+\Gamma_{j,n})^2}{4}}, \tag{9}$$

and (ii) a model with Fano resonances, in which

$$\tau_j(E, V_{sg,j}) = \sum_n \frac{\gamma_{j,n}\Gamma_{j,n}}{\left(E - \varepsilon_{j,n}(V_{sg,j}) - \frac{|t_{j,n}|^2}{E-\varepsilon'_{j,n}(V_{sg,j})}\right)^2 + \frac{(\gamma_{j,n}+\Gamma_{j,n})^2}{4}}. \tag{10}$$

Here $t_{j,n}$ is the hopping amplitude between the energy level $\varepsilon_{j,n}$, which is coupled to the leads, and to one of the closely lying uncoupled (dark) levels with the energy $\varepsilon'_{j,n}$. Of course, for some peaks one can have $t_{j,n} = 0$, and in this case the transmission has the same Lorentzian shape as in Supplementary Equation (9).

According to Supplementary Equation (7), the zero-bias dimensionless conductance of the dot in the zero temperature limit has the form

$$g_j(V_{sg,j}) = \frac{h}{e^2}\frac{dI_j}{dV_j}\bigg|_{V_j=0} = 2\tau(0, V_{sg,j}). \tag{11}$$

In the limit $k_BT_j, k_BT_S \ll \Gamma_{j,n} + \gamma_{j,n}$ and for $V_j = 0$, Supplementary Equation (7) reproduces the Mott formula for thermoelectric currents

$$I_j = \frac{\pi^2}{3}\frac{ek_B^2}{h}\frac{\partial\tau(0, V_{sg,j})}{\partial E}(T_j^2 - T_S^2). \tag{12}$$

The derivatives of the transmission probabilities over the energy E can be converted into the derivatives over the gate potentials if one uses Supplementary Equations (9,10) in combination with the gate efficiency relations in Supplementary Equation (6). In this way, one finds

$$I_j = -\frac{\pi^2}{6}\frac{ek_B^2}{a_jh}\frac{\partial g(V_{sg,j})}{\partial V_{sg,j}}(T_j^2 - T_S^2). \tag{13}$$

The corresponding thermopower, or the Seebeck coefficient, is given by

$$\alpha_j = \lim_{T_j\to T_S}\frac{\Delta V_j}{T_j - T_S}\bigg|_{I_j=0} = \lim_{T_j\to T_S}\frac{hI_j}{e^2g_j(V_{sg,j})(T_j - T_S)}\bigg|_{V_j=0} = \frac{\pi^2}{3}\frac{k_B^2T_S}{ea_j}\frac{1}{g_j(V_{sg,j})}\frac{\partial g_j(V_{sg,j})}{\partial V_{sg,j}}. \tag{14}$$

Supplementary Equations (13, 14) allow us to estimate the expected values of thermoelectric currents and the thermopower once the dependence of the zero bias conductance on the gate voltage has been measured. Supplementary Equation (13) also determines the sign of the thermoelectric current and the values of the gate voltage at which it equals to zero.

B. Local electric current in the superconducting state

In this section, we assume that the central lead of the system shown in Supplementary Fig. 7 is superconducting. In this case, the local transport properties of the individual dots are described by the theory of Andreev reflection at NS interfaces [10, 11], which needs to be generalized to the case of energy dependent transmission probability. For the resonant tunneling model, such generalization has been presented, for example, in Ref. 12. The result of Ref. 12 can be transformed to the form

$$\begin{aligned} I_j^{\rm loc}(E, V_{sg,j}) = \frac{e}{\pi\hbar}\int_{|E|<\Delta} dE \frac{\Delta^2[f_j(E - eV_j) - f_j(E + eV_j)]}{(\Delta^2 - E^2)\left[\left(\frac{2}{\tau_j(\tilde{E}_j, V_{sg,j})} - 1\right)\left(\frac{2}{\tau_j(-\tilde{E}_j, V_{sg,j})} - 1\right) - \frac{4\tilde{E}_j^2}{\Gamma_j^2(V_{sg,j})}\right] + E^2} \\ + \frac{2e}{\pi\hbar}\int_{|E|>\Delta} dE \frac{\nu_S(E)\left(\frac{2}{\tau(-E,V_{sg,j})} - 1 + \nu_S(E)\right)[f_j(E - eV_j) - f_S(E)]}{\left(\frac{2}{\tau(-E,V_{sg,j})} - 1 + \nu_S(E)\right)\left(\frac{2}{\tau(E,V_{sg,j})} - 1 + \nu_S(E)\right) + \frac{4\Delta^2}{\Gamma_j^2(V_{sg,j})}\nu_S^2(E)}, \end{aligned} \tag{15}$$

which is convenient for the analysis of the experimental data. Here we have introduced the re-normalized energy $\tilde{E}$ and the density of states in the superconductor $\nu_S(E)$,

$$\tilde{E}_j = E\sqrt{1 + \frac{\gamma_j(V_{sg,j})}{\sqrt{\Delta^2 - E^2}}}, \quad \nu_S(E) = \frac{|E|}{\sqrt{E^2 - \Delta^2}}. \tag{16}$$

We have also replaced the hopping rates of the individual levels $\Gamma_{j,n}$ and $\gamma_{j,n}$ by the gate-voltage-dependent expressions $\Gamma_j(V_{sg,j})$ and $\gamma_j(V_{sg,j})$. In fact, Supplementary Equation (15) provides a full description of the local transport through the quantum dots, and it can be used beyond the resonant tunneling model. For example, Supplementary Equation (15) accounts fully for the contribution of Andreev bound states, which are formed in the dots due to their coupling to a superconducting lead, and which have been observed in InAs [13] and in carbon nanotube quantum dots [14]. If the transmissions τ_j do not depend on the energy and $\Gamma_j \gg \Delta$, Supplementary Equation (15) reduces to well known result by Blonder Tinkham and Klapwijk [11]. Thermoelectric properties of a quantum point contact in this regime have been recently studied in detail in Ref. [15]. In the limit $\Delta = 0$, Supplementary Equation (15) takes the Landauer form (7). Finally, according to Supplementary Equation (15) in the low temperature limit, $k_B T_j, k_B T_S \ll \Delta, \Gamma_{j,n} + \gamma_{j,n}$, the zero bias conductance of the dot j acquires the form

$$g_j(V_{sg,j}) = \frac{h}{e^2} \frac{\partial I_j}{\partial V_j}\bigg|_{V_j=0} = \frac{4\tau_j^2(0, V_{sg,j})}{\left(2 - \tau_j(0, V_{sg,j})\right)^2}, \tag{17}$$

which, as expected, coincides with the prediction of the theory of Andreev reflection at low energies[10].

For an asymmetric quantum dot with low transmission, $\tau_j(E) \ll 1$, and for $eV, k_B T_j, k_B T_S, \Gamma_j, \gamma_j \ll \Delta$, Supplementary Equation (15) reduces to a simple expression

$$I_j^{\rm loc}(E, V_{sg,j}) = \frac{e}{2\pi\hbar} \int dE\, \tau_j(E, V_{sg,j})\tau_j(-E, V_{sg,j}) \left[f_j(E - eV_j) - f_j(E + eV_j)\right], \tag{18}$$

which contains the product of the transmission probabilities for an incoming electron, $\tau_j(E, V_{sg,j})$, and for a reflected hole, $\tau_j(-E, V_{sg,j})$.

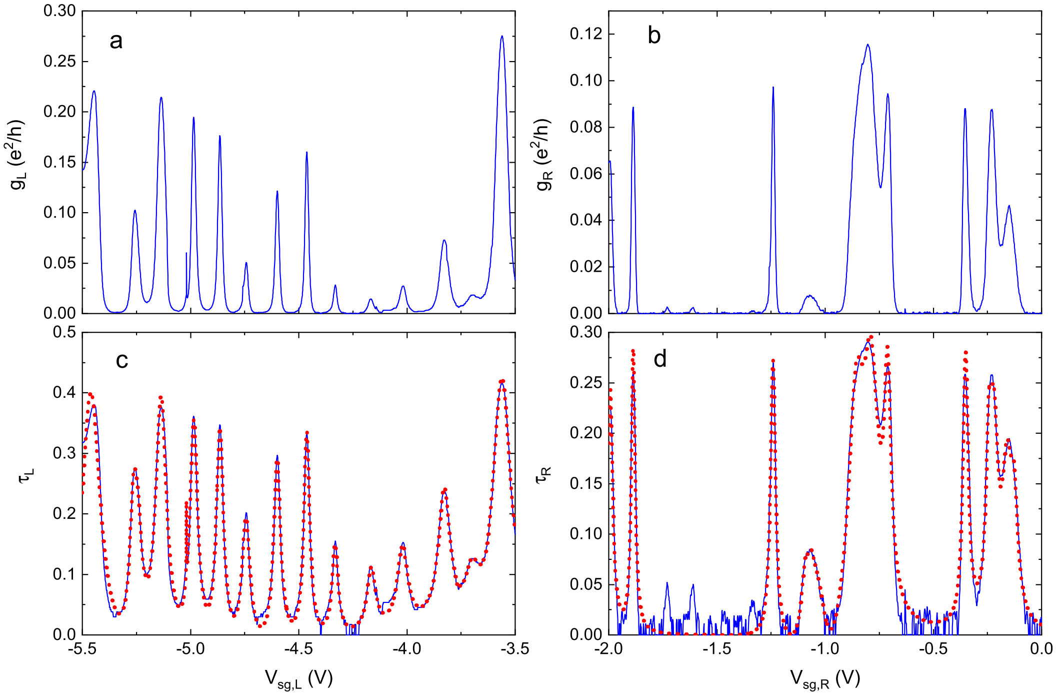

Supplementary Fig. 8. **Zero bias conductance of the quantum dots. a** and **b** — measured zero bias conductance of the left and right quantum dots. **c** and **d** — normal state transmission probabilities $\tau_L(V_{sg,L})$ and $\tau_R(V_{sg,R})$ obtained by applying the transformation of Supplementary Equation (19) to the experimental conductance curves depicted in panels **a** and **b**; red dotted lines indicate fits with multiple Lorentzian peaks with slowly varying background.

Supplementary Equation (17) in combination with the Lorentzian transmission probability of Supplementary Equation (9) has been demonstrated to reproduce accurately the shape of the conductance peaks in carbon nanotube quantum dots[14, 16]. We find that this model is also working quite well for our graphene quantum dots. In Supplementary Fig. 8a,b we display the zero-bias conductance of the left and right quantum dot, measured at $T_L = T_R = T_S = 90$ mK, as a function of the side gate voltages $V_{sg,L}$, $V_{sg,R}$. Inverting the expression (17) for zero bias conductance, we obtain normal state transmission probabilities in the form

$$\tau_j(0, V_{sg,j}) = \frac{2\sqrt{g_j(V_{sg,j})}}{2 + \sqrt{g_j(V_{sg,j})}}. \tag{19}$$

Applying this transformation to the experimental data sets depicted in Figs. 8 (a,b), we get the normal state transmission probabilities at zero energy $\tau_j(0, V_{sg,j})$. The latter are plotted in Figs. 8 (c,d) with blue lines. In the same figures, by red dotted lines we also show the fits of the transmissions $\tau_j(0, V_{sg,j})$ with multiple Lorentzian peaks in accordance with the predictions of the resonance tunneling model (Supplementary Equation (9)). The quality of these fits is rather good, especially for the left quantum dot. We conclude, therefore, that the resonance tunneling model describes the quantum dots well.

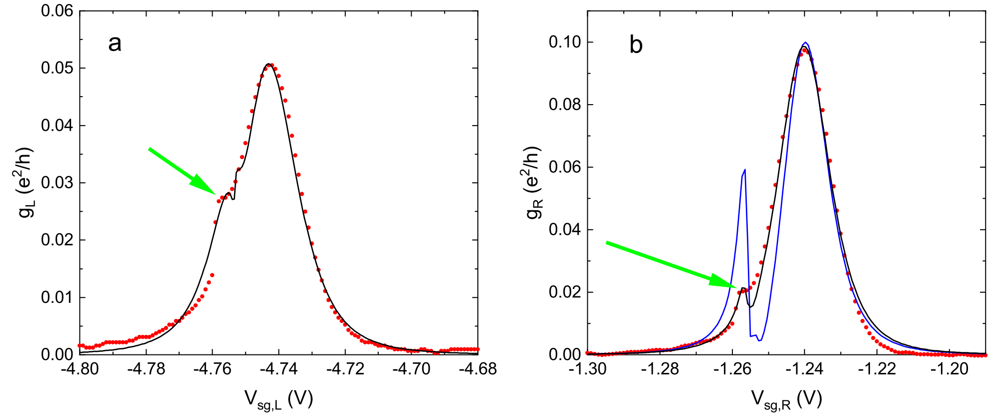

Supplementary Fig. 9. **Conductance fits.** Fits of the two selected zero bias conductance peaks of the left, **a**, and the right, **b**, quantum dots, measured at $T = 90$ mK, with Supplementary Equation (15), in which the transmission probabilities have the Fano resonance form (10). Green arrows indicate weak additional peaks not captured by the simple resonant tunneling model (9). **a** One of the conductance peaks of the left dot; red dots show experimental data, black is the fit to Supplementary Equations (10,15) with the parameters $\gamma_L = 98.3\,\mu$eV, $\Gamma_L = 6.04\,\mu$eV, $t_L = 10\,\mu$eV, $\varepsilon_L - \varepsilon'_L = 24\,\mu$eV. **b** Conductance peak of the right dot; red dots – experiment, black line is the best fit to Supplementary Equations (10,15) with the parameters $\gamma_R = 260.2\,\mu$eV, $\Gamma_R = 20.8\,\mu$eV $t_R = 22\,\mu$eV, $\varepsilon_R - \varepsilon'_R = 155\,\mu$eV, blue line shows the theoretical curve with the parameters used in Fig. 4 of the main text, namely, $\gamma_R = 252.4\,\mu$eV, $\Gamma_R = 20.4\,\mu$eV $t_R = 55\,\mu$eV, $\varepsilon_R - \varepsilon'_R = 120\,\mu$eV. The last set of parameters is used in Fig. 4 of the main text, in which the non-local contributions to the current are plotted.

A closer look at some of the conductance peaks reveals deviations from the form predicted by Supplementary Equation (17) with the Lorentzian transmission probabilities (9). In Supplementary Fig. 9 we plot two representative peaks, one for each dot, and indicate by the green arrows the features not captured using the resonant tunneling model. These features may be caused by various reasons. For example, they may result from Coulomb interaction, inelastic relaxation processes, or Kondo effect, etc. They may also arise from overlapping of the relevant energy levels. Here we fit these features using the Fano resonance model (10), in which a conductance peak splits into two closely lying peaks with different heights and widths. We believe that this is the most plausible explanation, although we cannot fully exclude other options. This uncertainty, however, does not affect the expression for the non-local current given below in Supplementary Equation (25) and in the main text. Indeed, the latter contains only the effective transmission probabilities τ_j, which can be inferred from the conductance data, and, therefore, are not very sensitive to a specific model describing the features of $\tau_j(E, V_{sg,j})$ and its parameter dependence. With the Fano resonance model, we have fitted the conductance peak of the left quantum dot quite accurately, see Fig 9a. However, for the right dot we have adopted a more pronounced Fano resonance than the conductance data would suggest (Fig 9b) in order to reproduce extra sign changes of the non-local thermoelectric current in the vicinity of this conductance peak (cf. Fig. 4 in the main text).

Having determined the zero-energy transmission probabilities of the quantum dots, $\tau_j(0, V_{sg,j})$, as functions of the gate voltages, we make the replacement $V_{gj} \to V_{gj} + E/\alpha_j$, where E is the energy of an electron, and in this way recover the full energy dependence of the transmission probabilities. Then, we use the obtained energy-dependent transmission probability $\tau_L(E, V_{sg,L})$ to calculate theoretical thermoelectric currents, based on Supplementary Equation (15), for different heating voltages. The result, displayed in Fig. 4a of the main text, agrees rather well with the experimental findings apart for the magnitude of the current. We would like to emphasize that, in this model, only quasiparticles with the energies $|E| > \Delta$ contribute to the thermoelectric current. That is why the predicted values of the current at low heating powers, $I_j \propto \exp(-\Delta/T_j)$, are very small. In the experiment, however, we observe significant thermoelectric currents even at the lowest heating voltages 1 mV $< V_h <$ 5 mV. This observation may be explained, for instance, by significant heating of the superconducting lead even at small heating voltages. Alternatively, it may be a result of the induced thermal voltages, which can be explained by the incoherent tunneling model discussed in Sec. C.

For completeness, we provide the low temperature expressions for the local thermoelectric currents, which follow from the

general expression of Supplementary Equation (15) for $V_L = V_R = 0$ and in the limit $T_L, T_R, T_S \ll \Delta/k_B$,

$$\begin{aligned} I_L^{\text{loc}} &= \frac{e\sqrt{2\pi\Delta}}{h}\,[\tau_L(\Delta) - \tau_L(-\Delta)]\Big[\sqrt{k_B T_L}\,e^{-\Delta/k_B T_L} - \sqrt{k_B T_S}\,e^{-\Delta/k_B T_S}\Big], \\ I_R^{\text{loc}} &= \frac{e\sqrt{2\pi\Delta}}{h}\,[\tau_R(\Delta) - \tau_R(-\Delta)]\Big[\sqrt{k_B T_R}\,e^{-\Delta/k_B T_R} - \sqrt{k_B T_S}\,e^{-\Delta/k_B T_S}\Big]. \end{aligned} \tag{20}$$

C. Non-local contributions to the thermoelectric current

The main focus of our study is Cooper pair splitting (CPS). It results from the crossed Andreev reflection process, in which a single Cooper pair splits into two electrons injected into different quantum dots. It has been shown (see e.g. Refs. 17–20) that the probability of such a process is proportional to the product of the transmission probabilities of the two quantum dots, $\tau_{L,R}(E)$, and the effective transmission probability of the superconducting electrode τ_S,

$$\tau_{\text{CPS}}(E) = \tau_L(E)\tau_S\tau_R(-E). \tag{21}$$

Strictly speaking, Refs. 17–20 deal with metallic normal leads, in which the transmission probabilities τ_L, τ_R do not depend on the electron energy E. Correct energy dependence of these transmissions can be obtained by comparing the crossed Andreev reflection probability of Supplementary Equation (21) with the probability of ordinary Andreev reflection, in which both electrons are emitted in to the same quantum dot. The latter probability appears in Supplementary Equation (18) for the Andreev current through a low transmission quantum dot and contains the product $\tau_j(E)\tau_j(-E)$, in which an electron and a reflected hole have opposite energies. Clearly, the same rule should apply to the crossed Andreev reflection, and in this way one arrives at the symmetry of Supplementary Equation (21). The effective transmission probability of the superconductor, τ_S, does not depend on the energy E if the quantum dots are placed close to each other and the distance between them is less than the coherence length of the superconductor. This condition is approximately satisfied in our experimental configuration. The parameter τ_S has the meaning of the probability for an electron emitted from one of the dots to reach the other dot instead of flying away into the bulk of the superconductor. From the theory of disordered superconductors based on the Usadel equation one formally finds [19]

$$\tau_S \approx \frac{4\pi\hbar R_S}{e^2}e^{-d/\xi_S}, \tag{22}$$

where R_S is the resistance of the superconducting lead in the normal state, d is the distance between the two dots, $\xi_S = \sqrt{D/2\Delta}$ is the coherence length inside the superconductor and $D = v_F L_e/3$ is the diffusion constant. Supplementary Equation (22) is valid if the electron mean free path l_e is much smaller than the distance between the dots. In practice, the value of τ_S is sensitive to the sample geometry, granularity of the superconductor, quality of the contacts between the superconductor and the quantum dots, etc. Therefore, we treat it as a fit parameter. We find that $\tau_S = 0.1$ provides good fit of our data.

It is well known that in addition to the Cooper pair splitting process, elastic cotunneling also contributes to the non-local transport. The probability of this process is given by the product [17–20]

$$\tau_{\text{EC}}(E) = \tau_L(E)\tau_S\tau_R(E), \tag{23}$$

which differs from τ_{CPS} only by the replacement $E \to -E$ in the argument of τ_R. The approximate expressions in Supplementary Equations (21,23) are valid provided $\tau_{\text{CPS}}, \tau_{\text{EC}} \ll 1$. This condition is well satisfied in our experiment.

The currents flowing through the quantum dots are given by the sum of local and non-local contributions,

$$I_L = I_L^{\text{loc}} + \Delta I_L^{\text{nl}}, \quad I_R = I_R^{\text{loc}} + \Delta I_R^{\text{nl}}, \tag{24}$$

where the local currents are given by Supplementary Equation (15), and the non-local corrections in the low temperature limit $k_B T_L, k_B T_R \ll \Delta$ and zero voltage drops across the dots, $V_j = 0$, are expressed as

$$\begin{aligned} \Delta I_L^{\text{nl}} &= \frac{e\tau_S}{h}\int dE\,\tau_L(E)[\tau_R(E) + \tau_R(-E)]\left[\frac{1}{1+e^{E/k_B T_L}} - \frac{1}{1+e^{E/k_B T_R}}\right], \\ \Delta I_R^{\text{nl}} &= -\frac{e\tau_S}{h}\int dE\,[\tau_L(E) + \tau_L(-E)]\tau_R(E)\left[\frac{1}{1+e^{E/k_B T_L}} - \frac{1}{1+e^{E/k_B T_R}}\right]. \end{aligned} \tag{25}$$

One can also define the linear combinations of the non-local currents, which are determined solely by Cooper pair splitting or elastic cotunneling,

$$\begin{aligned} \Delta I_{\text{CPS}} &= \Delta I_L^{\text{nl}} + \Delta I_R^{\text{nl}} = \frac{2e}{h}\int dE\,\tau_{\text{CPS}}(E)\left[\frac{1}{1+e^{E/k_B T_L}} - \frac{1}{1+e^{E/k_B T_R}}\right], \\ \Delta I_{\text{EC}} &= \Delta I_L^{\text{nl}} - \Delta I_R^{\text{nl}} = \frac{2e}{h}\int dE\,\tau_{\text{EC}}(E)\left[\frac{1}{1+e^{E/k_B T_L}} - \frac{1}{1+e^{E/k_B T_R}}\right]. \end{aligned} \tag{26}$$

These expressions presented and discussed in the main text.

In order to understand the overall behaviour of the non-local currents, we again consider the low temperature limit $k_BT_L, k_BT_R \ll \Delta, \gamma_{j,n} + \Gamma_{j,n}$. In this limit one can derive the expressions analogous to Mott's formula (13),

$$\Delta I_L^{\rm nl} = \frac{\pi^2}{3}\frac{ek_B^2}{h}\frac{\partial\tau_L(0,V_{sg,L})}{\partial E}\tau_S\,\tau_R(0,V_{sg,R})(T_L^2 - T_S^2)$$
$$= -\frac{\pi^2}{3}\frac{ek_B^2}{a_L h}\tau_S\frac{\partial g_L(0,V_{sg,L})}{\partial V_{sg,L}}\frac{4\sqrt{g_R(V_{sg,R})}}{\sqrt{g_L(V_{sg,L})}\left(2+\sqrt{g_L(V_{sg,L})}\right)^2\left(2+\sqrt{g_R(V_{sg,R})}\right)}(T_L^2 - T_S^2), \quad (27)$$

$$\Delta I_R^{\rm nl} = \frac{\pi^2}{3}\frac{ek_B^2}{h}\tau_L(0,V_{sg,L})\tau_S\frac{\partial\tau_R(0,V_{sg,R})}{\partial E}(T_R^2 - T_S^2)$$
$$= -\frac{\pi^2}{3}\frac{ek_B^2}{a_R h}\tau_S\frac{\partial g_R(0,V_{sg,R})}{\partial V_{sg,R}}\frac{4\sqrt{g_L(V_{sg,L})}}{\sqrt{g_R(V_{sg,R})}\left(2+\sqrt{g_L(V_{sg,L})}\right)\left(2+\sqrt{g_R(V_{sg,R})}\right)^2}(T_R^2 - T_S^2). \quad (28)$$

Supplementary Equation (27), for example, shows that the non-local contribution to the thermoelectric current of the left quantum dot should change sign at the values of the gate voltage $V_{sg,L}$ corresponding to the positions of the conductance peaks and the minima of the conductance valleys of the left dot. At the same time, no sign change in $\Delta I_L^{\rm nl}$ occurs if one varies the gate potential of the right dot $V_{sg,R}$. The current $\Delta I_L^{\rm nl}$ depends on the right gate potential $V_{sg,R}$ roughly in the same way as the conductance of the right dot $g_R(V_{sg,R})$ does. Thus, in order to restore the sign pattern and relative magnitudes of the thermoelectric currents one can use a simple rule $\Delta I_L^{\rm nl} \propto (dg_L/dV_{sg,L})g_R$, $\Delta I_R^{\rm nl} \propto g_L(dg_R/dV_{sg,R})$. Such Mott-type behaviour of the non-local currents agrees well with gate voltage dependence observed in the experiment. Similar sign pattern is predicted by a slightly different model, in which a quantum dot is coupled to two normal and superconducting terminals [21].

SUPPLEMENTARY NOTE 5: THEORETICAL MODELING: INCOHERENT TRANSPORT IN A N-DOT-S-DOT-N SYSTEM.

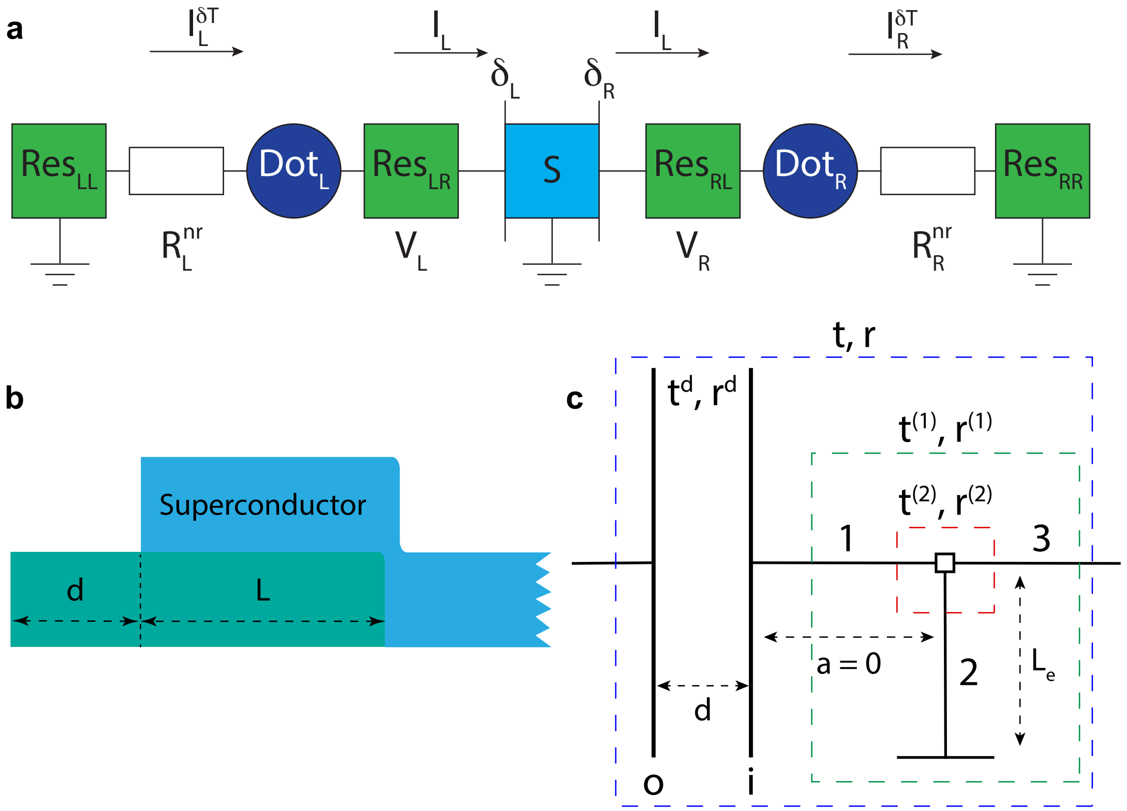

Supplementary Fig. 10. **Incoherent model.** **a** Schematics of the experimental device in the incoherent model. Res_{LL}, Res_{LR}, Res_{RL}, and Res_{RR} are the electron-reservoirs, $Dot_{L(R)}$ is the left (right) quantum dot, R_L^{nr} and R_R^{nr} are the additional resistors, S is the superconducting region, $\delta_{L(R)}$ is δ-barrier on the left (right) NS-interface. Res_{LL}, Res_{RR} and S are grounded. **b** Side view (schematic) of the graphene-aluminum (superconductor) junction. The patterned graphene piece has a characteristic size of $\sim$ 300 nm and constitutes a quantum dot. Here L and d are the lengths of the graphene parts which, respectively, are either overlapping or extending beyond the superconductor. **c** Schematics of the theoretical model accounting for the effect of the additional Fano resonance structure. The quantum dot is modelled by two δ-function potentials (the distance between barriers is d) connected to a fork with a stub of length $L_e \sim L^2/\lambda$. The transmission and reflection amplitudes of the beam splitter (blank square) are denoted by $t^{(2)}$ and $r^{(2)}$; the amplitudes incorporating the internal features of the fork and the stub are denoted by $t^{(1)}$ and $r^{(1)}$. We set the distance a between the double barrier and the beam splitter equal to zero.

The model described in Supplementary Note 4 is based on the assumption that the transport in the Dot-S-Dot system is fully coherent. In reality, the system may be subject to dephasing and relaxation. To demonstrate how the decoherence processes may affect the experiment, we present and analyze an alternative theoretical model based on the scattering matrix approach. This model is predicated on the assumption that the system may be split into the coherent subsystems which, in turn, are joined incoherently as connected circuit elements. For this reason, thermal gradients induce both electric currents and voltage drops on the dots. That is why, in contrast to the coherent transport model, the incoherent one predicts significant value of the local thermoelectric currents at low temperatures, where the quasiparticles in the superconducting lead disappear, and, thus, it may explain the experimental observations in this regime. However, our analysis shows that decoherence has little effect on the measured non-local phenomena.

To account for possible decoherence, we assume that the schematics includes additional reservoirs (Res_{LR} and Res_{RL}) between the superconductor and both the left and the right dot as depicted in Supplementary Fig. 10a. Similar models of decoherence have been considered elsewhere, see e.g. Ref. [22]. We model the whole structure as a one-dimensional conducting structure with ballistic motion of electrons in the superconductor and in the quantum dots. The superconductor (S) is separated by δ-barriers (δ_L and δ_R) from the adjacent reservoirs. The transmission probabilities of the quantum dots (Dot_L and Dot_R) may exhibit Fano resonances. In order to model such resonances, the dots are assumed to be composed of two elements — Fabry-Pérot double barrier structure and a stub. Finally, we assume that the leftmost and rightmost reservoirs (Res_{LL} and Res_{RR}) along with the superconductor are grounded.

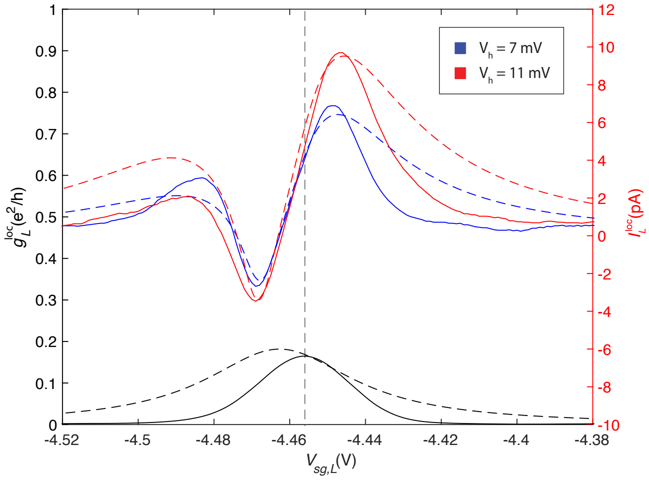

Supplementary Fig. 11. **Local thermoelectricity.** Uppermost curves: fits (dashed) of the experimentally measured local thermoelectric currents (solid) at two different heating voltages V_h (7 and 11 mV). Lower traces: experimentally measured (solid) and theoretically modelled (dashed) conductance. The parameters are $t_{13}^{(2)} = 0.01\, e^{-0.03i}$, $t_{23}^{(2)} = 0.55$, $Z_o = 5$, $Z_i = 5$, $R = 5\frac{h}{e^2}$, $2k_F L_e = 2.33 + 2\pi n$ (n is integer), $d = 2.0$ nm, $L_e = 1810.0$ nm (we assume that the Fermi velocity in graphene is $v_F = 10^6 m/s$). We take $T_{LL} = (0.09 + 9.1\, V_h^{0.70})$ K and $T_{LR} = (0.09 + 8.67\, V_h^{0.70})$ K.

A. Local electric current

Let us fist discuss the characteristic features of the experimental plots showing the dependence of the local thermoelectric current on the gate voltage in the quantum dot. For the present model we assume that the local thermoelectricity in the left (right) side of the structure is governed by the difference between the population distributions in $\mathrm{Res_{LL}}$ and $\mathrm{Res_{LR}}$ ($\mathrm{Res_{RL}}$ and $\mathrm{Res_{RR}}$) due to the temperature gradient. For simplicity, we will consider only the subgap regime.

As mentioned in the main paper, we can explain the extraordinary behavior of the local thermoelectric current (i.e., the appearance of a secondary extremum on one side of the main inflexion point) by the Fano resonant effect emerging in the connection between the dot and the superconductor. The arrangement of the experimental setting (as shown in Supplementary Fig. 10b) suggests that it can be modeled by the scheme depicted in Supplementary Fig. 10c. Here the interface between the superconductor and the double delta barrier (the distance between barriers is d) is considered to have a fork structure with one of its contacts being a stub of effective length L_e. L_e is much larger than d which ensures the interaction between the discreet spectrum due to the stub and the semi-continuum in the double delta barrier (which encompasses a large level width). Drawing a parallel with the schematics in Supplementary Fig. 10b, the stub relates to the part of graphene overlapping with the superconductor, which has length L. The length of the stub L_e, however, should not necessarily be equal to L: since the mean free path λ of the particles is smaller than L, in reality they may undergo multiple reflections inside this part of the structure. Then

$$L^2 \sim D t_t, \tag{29}$$

where $D \sim v_F \lambda$ is the diffusion coefficient and t_t is the time that the particle spends in the stub. Accordingly, the effective length of the stub $L_e \sim v_F t_t \sim L^2/\lambda$ used in the model can exceed the typical size of the dot $L \simeq 300$ nm. We assume that the barrier in the end of the stub completely blocks the propagation of the particles, i.e., the probability density for finding the particle beyond the barrier is zero. For clarity, we separate the right delta barrier from the beam splitter, but imply that the distance a between them is zero. If L_e is finite, the appearance of Fano resonances affects the transparency between left and right terminals changing the behaviour of the thermoelectric current.

Next we derive the transparency function which would account for the occurrence of this additional Fano scattering in the dot structure.

Double delta barrier.– The amplitudes of the transmission and reflection (from the inner delta barrier) associated with the double barrier can be written in the form

$$t^d = t_i t_o e^{ik_d d}/(1 - r_i r_o e^{2ik_d d}); \tag{30}$$

$$r_i^d = r_i + r_o t_i^2 e^{2ik_d d}/(1 - r_i r_o e^{2ik_d d}); \tag{31}$$

where $t_{i(o)} = 1/(1 + iZ_{i(o)})$ and $r_{i(o)} = -iZ_{i(o)}/(1 + iZ_{i(o)})$ are the transmission and reflection amplitudes of the inner (outer) delta barrier (expressed in terms of strengths Z_i and Z_o), d is the distance between delta barriers and

$$k_d = k_F + \frac{E - eV_g}{\hbar v_F} \tag{32}$$

is the wave vector of the particle inside the double barrier (here, V_g is the voltage on the gate).

Fork.– Supposing that the wave function of the particles goes to zero on the boundary in the stub and the stub is grounded (i.e., it is not affected by the gate voltage on the dot), we may obtain the transmission and reflection amplitudes $t^{(1)}$, $r^{(1)}$ on the contact 1-3 (incorporating the internal features, i.e., the stub, see Supplementary Fig. 10c):

$$t_{13}^{(1)} = t_{13}^{(2)} - \frac{t_{12}^{(2)} t_{23}^{(2)}}{\exp(-2ikL_e) + r_{22}^{(2)}}, \tag{33}$$

$$r_{33}^{(1)} = r_{33}^{(2)} - \frac{t_{32}^{(2)} t_{23}^{(2)}}{\exp(-2ikL_e) + r_{22}^{(2)}}, \tag{34}$$

$$r_{11}^{(1)} = r_{11}^{(2)} - \frac{t_{12}^{(2)} t_{21}^{(2)}}{\exp(-2ikL_e) + r_{22}^{(2)}}, \tag{35}$$

where index (2) denotes the transmission and reflection amplitudes of the beam splitter, and L_e is the length of the stub; we assume that the wave vector $k = k_F + \frac{E}{\hbar v_F}$ in the stub does not depend on the gate voltage.

For the numerical calculations we will parametrize the beam splitter's scattering matrix with only two parameters: the elements of the matrix can be expressed in terms of the transmission probabilities in contacts 1-3 and 2-3:

$$\tau_{12}^{(2)} = \tau_{13}^{(2)} \tau_{23}^{(2)} \left[2 - \tau_{\Sigma}^{(2)} + 2 \left(1 - \tau_{\Sigma}^{(2)} \right)^{1/2} \right] / (\tau_{\Sigma}^{(2)})^2, \tag{36}$$

$$r_{ii}^{(2)} = t_{jk}^{(2)*} t_{ij}^{(2)} t_{ik}^{(2)} \left[(\tau_{jk}^{(2)})^{-1} - (\tau_{ij}^{(2)})^{-1} - (\tau_{ik}^{(2)})^{-1} \right] /2, \tag{37}$$

where $\tau_{\Sigma}^{(2)} \equiv \tau_{13}^{(2)} + \tau_{23}^{(2)}$, and $\tau_{ij}^{(2)} = \left| t_{ij}^{(2)} \right|^2$.

Whole structure.– The final transmission probability $\tau = |t|^2$ of the dot can be calculated by substituting Supplementary Equations (30-36) into the formula

$$t = \frac{t^d t_{13}^{(1)}}{1 - r_{11}^{(1)} r_i^d}. \tag{38}$$

The scattering on the outer (O) and inner (I) points of the structure is described by the reflection amplitudes

$$r_I = r_{33}^{(1)} + \frac{r_i^d (t_{13}^{(1)})^2}{1 - r_{11}^{(1)} r_i^d}; \tag{39}$$

$$r_O = r_o^d + \frac{r_{11}^{(1)} (t^d)^2}{1 - r_{11}^{(1)} r_i^d}. \tag{40}$$

In the experiment, the local conductance and current are measured in the network of the dot along with the graphene nanoribbon (represented by $R_{L(R)}^{\mathrm{nr}}$ in Supplementary Fig. 10a) and the N-δ-S boundary. For the theoretical model, we should assume that the nanoribbon, together with the N-δ-S boundary, have a constant electrical resistance R. This allows us to write the following equation for the local current through the left dot (the transparency of the dot τ_L is calculated as discussed above):

$$I_L^{\mathrm{loc}} = \frac{2e}{h} \int dE \left[\frac{1}{\exp \frac{E + e(-V + I_L^{\mathrm{loc}} R)\alpha}{k_B T_{LL}} + 1} - \frac{1}{\exp \frac{E + e(-V + I_L^{\mathrm{loc}} R)(\alpha - 1)}{k_B T_{LR}} + 1} \right] \tau_L(E), \tag{41}$$

where V denotes the external voltage applied to the system (we will need V later to calculate the differential conductance), $T_{LL(LR)}$ is the temperature of $\mathrm{Res}_{LL(LR)}$ and α $(0 \leq \alpha \leq 1)$ describes the voltage distribution across the dot. Since $\delta T_L = T_{LL} - T_{LR} \ll T_{LR}$ and $eI_L^{\mathrm{loc}} R \ll k_B T_{LR}$ (as one will be able to see later), we obtain

$$I_L^{\mathrm{loc}} = \frac{2e}{h} \int dE \frac{\partial f}{\partial E} [-eV + eI_L^{\mathrm{loc}} R - E \delta T_L / T_{LR}] \, \tau_L(E), \tag{42}$$

whence

$$I_L^{\rm loc} = \frac{-\frac{2e}{h}\int dE\frac{\partial f}{\partial E}[eV + E\delta T_L/T_{LR}]\,\tau_L(E)}{1 - R\cdot\frac{2e^2}{h}\int dE\frac{\partial f}{\partial E}\tau_L(E)}. \quad (43)$$

The parameters of our model are such that the dependence of $\tau_L(E)$ on E is relatively weak. We can therefore write the following equations for the current $I_L^{\rm loc}$ ($V = 0$) and the differential conductance $g_L^{\rm loc} = \frac{\partial I_L^{\rm loc}}{\partial V}$:

$$I_L^{\rm loc} = \frac{2\pi^2 e k_B^2 T_{LR}\delta T_L}{3h}\frac{\partial\tau_L/\partial E(0)}{1 + \frac{2e^2}{h}\tau_L(0)R}; \quad (44)$$

$$g_L^{\rm loc} = \frac{2e^2}{h}\frac{\tau_L(0)}{1 + \frac{2e^2}{h}\tau_L(0)R}. \quad (45)$$

Supplementary Fig. 11 depicts experimental fits for different heating voltages V_h obtained using Supplementary Equations (36), (38) and (44); we put temperatures $T_{LL} = (0.09 + 9.1\,V_h^{0.70})$ K and $T_{LR} = (0.09 + 8.67\,V_h^{0.70})$ K; other parameters are given in the caption. One can see that, for small chosen values of $\tau_{13}^{(2)}$, the behaviour of the thermoelectric current predicted theoretically compares well with the experimental data. Moreover, we should note that the parameters of the fits are such that the length $L_e = 1810$ nm comply with our prediction of $\sim L^2/\lambda \sim 2000$ nm given that $L \simeq 300$ nm and $\lambda \sim 30$ nm. Thus, the above model, accounting for quasi-particles in the superconductor at elevated temperatures, allows for a faithful theoretical description of the measured data. However, the model does not reproduce the experimental observation that the secondary features of the curves tend to disappear at large heating voltages.

B. Non-local electric current

The non-local currents in this model are contributed by two factors:

- The voltage difference between $\mathrm{Res_{LR}}$ and $\mathrm{Res_{RL}}$ which, in turn, is induced by the local thermoelectricity in the left and right leads. For instance, the temperature difference between $\mathrm{Res_{LL}}$ and $\mathrm{Res_{LR}}$ generates a nonzero electric potential in $\mathrm{Res_{LR}}$.
- The temperature gradient between $\mathrm{Res_{LR}}$ and $\mathrm{Res_{RL}}$.

Our numerical analysis shows that the latter factor does not play a significant role and can be neglected. Physically, this is due to the fact that the transmission through the $\delta_{\rm L}$-S-$\delta_{\rm R}$ structure depends on the energy of the incident particles E rather weakly compared to the transmission through the dots.

The left-to-right current in the left lead, which flows from Res_{LR} to S and Res_{RL} is given by [23]

$$I_L(T_{LR}, T_{RL}, V_L, V_R) = \frac{2e}{h}\int dE\,\{-\mathcal{R}_{eh}^{LL}(E)\,[1 - f(E - eV_L, T_{LR})] - \mathcal{T}_{eh}^{RL}(E)\,[1 - f(E - eV_R, T_{RL})] \\ + [1 - \mathcal{R}_{ee}^{LL}(E)]\,f(E - eV_L, T_{LR}) - \mathcal{T}_{ee}^{RL}(E)\,f(E - eV_R, T_{RL})\}, \quad (46)$$

where $T_{LR(RL)}$ and $V_{L(R)}$ are, respectively, the temperature and electric potential of $\mathrm{Res}_{LR(RL)}$, $\mathcal{R}_{eh(ee)}^{LL}(E)$ is the probability of Andreev (normal) reflection on the N-δ_L-S interface, $\mathcal{T}_{eh(ee)}^{RL}$ is the probability of the Cooper pair splitting (elastic co-tunneling) with regard to the particles incident from the right lead (here the upper subscript indicates the direction of the particle motion; e.g., RL means that the particle incident from the right lead is transmitted into the left one).

The probabilities $\mathcal{T}_{eh(ee)}^{LL} = |\tilde{r}_{eh(ee)}|^2$ and $\mathcal{R}_{eh(ee)}^{RL} = |\tilde{t}_{eh(ee)}|^2$ are determined by the transmission and reflection amplitudes given by [24]

$$\tilde{t}_{eh} = t_L\,[t_{ee}\,r_R\,r_{eh} + r_{eh}\,r_L\,t_{hh}]\,t_R/\mathcal{D}; \quad (47)$$

$$\tilde{t}_{ee} = t_L\,[t_{ee}\,(1 - t_{hh}^2\,r_L\,r_R) + r_{eh}\,r_L\,t_{hh}\,r_R\,r_{he}]\,t_R/\mathcal{D}; \quad (48)$$

$$\tilde{r}_{eh} = t_L\,r_{eh}\,[1 + (t_{ee}\,t_{hh} - r_{eh}\,r_{he})\,r_R\,r_R]\,t_L/\mathcal{D}; \quad (49)$$

$$\tilde{r}_{ee} = r_L + t_L\,[r_{eh}\,r_L\,r_{he} + t_{ee}\,r_R\,t_{ee} - (t_{ee}\,t_{hh} - r_{eh}\,r_{he})^2\,r_L\,r_R\,r_R]\,t_L/\mathcal{D}, \quad (50)$$

where $t_{L(R)}$ and $r_{L(R)}$ are the transmission and reflection amplitudes of $\delta_{L(R)}$; $\mathcal{D}$ is determined by multiple reflections inside the δ_L-S-δ_R structure:

$$\mathcal{D} = 1 - t_{ee}^2\,r_L\,r_R - t_{hh}^2\,r_L\,r_R - r_{eh}\,r_{he}\,(r_L\,r_L + r_R\,r_R) + (t_{ee}\,t_{hh} - r_{eh}\,r_{he})^2\,r_L\,r_R\,r_L\,r_R; \quad (51)$$

and $t_{ee(hh)}$ and $r_{ee(hh)}$ are the trasmission and reflection amplitudes of the superconducting part of the structure:

$$t_{ee(hh)} = \frac{e^{\pm ipl_S}\sin\alpha}{\sin(\alpha - iql_S)}; \tag{52}$$

$$r_{eh(he)} = \frac{\sinh ql_S}{i\sin(\alpha - iql_S)}. \tag{53}$$

Here $p^2 - q^2 = k_F^2$ and $2pq = (2m/\hbar^2)\Delta\sin\alpha$ with $\alpha = \arccos(E/\Delta)$; l_S is the size of the superconducting region.

Bearing in mind that $I_L(T, T, 0, 0) = 0$ and that the temperature difference between Res_{LR} and Res_{RL} has a small contribution to the thermoelectric current, from Supplementary Equation (46) we obtain

$$\begin{aligned} I_L(T_{LR}, T_{RL}, V_L, V_R) &\simeq \frac{2e^2}{h}\int dE\,\{-\mathcal{R}_{eh}^{LL}(E)\,\frac{\partial f}{\partial E}(E, T_{LR})\,V_L - \mathcal{T}_{eh}^{RL}(E)\,\frac{\partial f}{\partial E}(E, T_{RL})\,V_R \\ &\quad - [1-\mathcal{R}_{ee}^{LL}(E)]\,\frac{\partial f}{\partial E}(E, T_{LR})\,V_L + \mathcal{T}_{ee}^{RL}(E)\,\frac{\partial f}{\partial E}(E, T_{RL})\,V_R \\ &\simeq \frac{2e^2}{h}[2\mathcal{R}_{eh}^{LL}(0) + 2\mathcal{T}_{eh}^{LR}(0)]\,V_L + \frac{2e^2}{h}[\mathcal{T}_{ee}^{LR}(0) - \mathcal{T}_{eh}^{LR}(0)]\,V_L - \frac{2e^2}{h}[\mathcal{T}_{ee}^{RL}(0) - \mathcal{T}_{eh}^{RL}(0)]\,V_R \\ &= G_L^{NS}\,V_L + G_L^{NSN}\,V_L - G_R^{NSN}\,V_R, \end{aligned} \tag{54}$$

where the quantities defined as

$$G_{L(R)}^{NS} = \frac{2e^2}{h}[2\mathcal{R}_{eh}^{LL(RR)}(0) + 2\mathcal{T}_{eh}^{LR(RL)}(0)], \tag{55}$$

$$G_{L(R)}^{NSN} = \frac{2e^2}{h}[\mathcal{T}_{ee}^{LR(RL)}(0) - \mathcal{T}_{eh}^{LR(RL)}(0)] \tag{56}$$

may be said to represent, respectively, N-δ-S and N-δ-S-δ-N conductances. Similarly, the left-to-right current in the right lead, flowing from S and Res_{LR} to Res_{RL} is given by

$$\begin{aligned} I_R(T_{LR}, T_{RL}, V_L, V_R) &= -\frac{2e}{h}\int dE\,\{-\mathcal{R}_{eh}^{RR}(E)\,[1 - f(E - eV_R, T_{RL})] - \mathcal{T}_{eh}^{LR}(E)\,[1 - f(E - eV_L, T_{LR})] \\ &\quad + [1 - \mathcal{R}_{ee}^{RR}(E)]\,f(E - eV_R, T_{LR}) - \mathcal{T}_{ee}^{LR}(E)\,f(E - eV_L, T_{LR})\} \\ &\simeq -G_R^{NS}\,V_R + G_L^{NSN}\,V_L - G_R^{NSN}\,V_R. \end{aligned} \tag{57}$$

Assuming now that δ_L and δ_R are identical, we have $G_L^{NS} = G_R^{NS} \equiv G^{NS}$ and $G_L^{NSN} = G_R^{NSN} \equiv G^{NSN}$. Thus,

$$I_L = G^{NS}\,(V_L - 0) + G^{NSN}\,(V_L - V_R); \tag{58}$$

$$I_R = G^{NS}\,(0 - V_R) + G^{NSN}\,(V_L - V_R). \tag{59}$$

Using Kirchhoff's law for the currents at the intersection of Res_{LR}, Res_{RL}, and the superconductor, we obtain the following equations:

$$I_L^{\delta T} + g_L\,(0 - V_L) = G^{NS}\,(V_L - 0) + G^{NSN}\,(V_L - V_R); \tag{60}$$

$$I_R^{\delta T} + g_R\,(V_R - 0) = G^{NS}\,(0 - V_R) + G^{NSN}\,(V_L - V_R), \tag{61}$$

where $g_{L(R)}$ is the conductance of $\mathrm{Dot}_{L(R)}$ plus additional resistor (nanoribbon) $R_{L(R)}^{\mathrm{nr}}$, and $I_{L(R)}^{\delta T}$ is the locally induced thermoelectric current:

$$g_{L(R)} = \left(\frac{1}{\frac{2e^2}{h}\tau_{L(R)}(0)} + R_{L(R)}^{\mathrm{nr}}\right)^{-1} = \frac{2e^2}{h}\,\frac{\tau_{L(R)}(0)}{1 + \frac{2e^2}{h}\tau_{L(R)}(0)R_{L(R)}^{\mathrm{nr}}}; \tag{62}$$

$$I_L^{\delta T} = \frac{2e}{h}\int dE\,\tau_L(E)\,(f(E, T_{LL}) - f(E, T_{LR})); \tag{63}$$

$$I_R^{\delta T} = \frac{2e}{h}\int dE\,\tau_R(E)\,(f(E, T_{RL}) - f(E, T_{RR})). \tag{64}$$

We thus obtain

$$V_R = \frac{-I_R^{\delta T} + I_L^{\delta T} G^{NSN}/(g_L + G^{NS} + G^{NSN})}{g_R + G^{NS} + G^{NSN} - (G^{NSN})^2/(g_L + G^{NS} + G^{NSN})}; \tag{65}$$

$$V_L = \frac{I_L^{\delta T} + G^{NSN} V_R}{g_L + G^{NS} + G^{NSN}}. \tag{66}$$

The currents can be found by substituting V_L and V_R into Supplementary Equation (58). To select the non-local contribution from the $I_{L(R)}$, one has to subtract $\langle I_{L(R)} \rangle$ (averaging should be performed over different gate voltages on the right (left) dot) from $I_{L(R)}$:

$$\Delta I_{L(R)}^{\text{nl}} = I_{L(R)} - \langle I_{L(R)} \rangle. \tag{67}$$

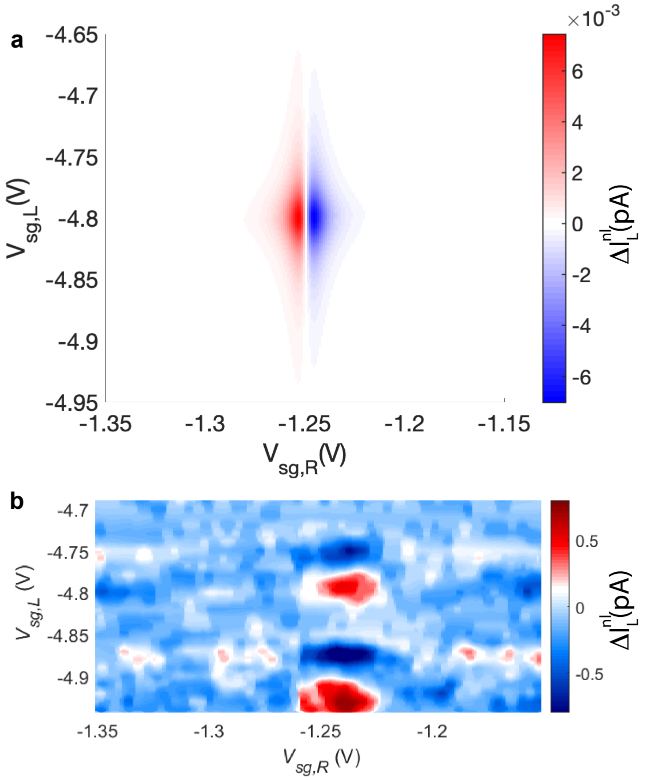

Supplementary Fig. 12. **Non-local thermoelectricity.** Non-local current ΔI_L^{nl} on the left side as function of the gate voltages $V_{sg,L}$ and $V_{sg,R}$ obtained **a** theoretically using the incoherent model and **b** experimentally. The parameters for **a** are such that the Fano resonance structure in the dots is absent: the parameters of both dots are $t_{13}^{(2)} = 1$, $t_{23}^{(2)} = 0$, $Z_o = 5$, $Z_i = 5$; $R_L^{\text{nr}} = R_R^{\text{nr}} = 5\frac{h}{e^2}$, $d = 2.0$ nm, the Fermi energy $E_F \gg \Delta$, $l_S = k_F \Delta/(2E_F)$, $pl_S = 0.9\pi + 2\pi n$ (n is integer); $t_L = t_R = 1/(1+i)$, $r_L = r_R = -i/(1+i)$ $T_{LL} = (0.09 + 9.1\,V_h^{0.70})$ K, $T_{LR} = (0.09 + 8.8\,V_h^{0.70})$ K, $T_{RL} = (0.09 + 8.8\,V_h^{0.70})$ K, $T_{RR} = (0.09 + 8.4\,V_h^{0.70})$ K.

As indicated by the calculations above, in the case of the incoherent system, the non-local electrical currents mostly originates from the electric potential difference between the intermediate reservoirs Res$_{LR}$ and Res$_{RL}$. Therefore, in a strict sense, $\Delta I_{L(R)}^{\text{nl}}$ is not thermoelectric current but rather it is produced by the locally induced thermoelectric voltages V_L and V_R on Res$_{LR}$ and Res$_{RL}$, which in this regard act as proxies. However, the plots for $\Delta I_{L(R)}^{\text{nl}}$ obtained from the present model are fundamentally different from the experimental data, which suggests that the observed non-local currents have a direct thermoelectric nature: Supplementary Fig. 12 displays the theoretically and experimentally obtained non-local current ΔI_L^{nl} on the left side as function of the gate voltages $V_{sg,L}$ and $V_{sg,R}$ in a simple situation where $\tau_L(E)$ and $\tau_R(E)$ have a Lorentzian form (i.e., the Fano resonance effects are absent). Both plots are characterized by similar patterns, but one can easily observe that they have different orientations of their symmetry axes. This indicates that while the experimental setup can still be subject to decoherence, which may enable the above discussed mechanism for the local thermoelectricity, the non-local current is mostly determined by the coherent electrical transport. Note that the exact values of $\Delta I_{L(R)}^{\text{nl}}$ in Supplementary Fig. 12b are not important since the purpose is rather to show the distinctive behaviour of the non-local electricity conditioned by the incoherent transport.

To conclude, the incoherent description accurately predicts the character of the local thermoelectricity at small temperatures. At the same time, at variance to effect of the local thermoelectricity, the non-local currents are dominantly determined by coherent electrical transport.

---

[1] Song, J. C. W., Reizer, M. Y. & Levitov, L. S. Disorder-assisted electron-phonon scattering and cooling pathways in graphene. *Phys. Rev. Lett.* **109**, 106602 (2012).

[2] Betz, A. C. *et al.* Supercollision cooling in undoped graphene. *Nat. Phys.* **9**, 109–112 (2012).

[3] Swartz, E. & Pohl, R. Thermal boundary resistance. *Rev. Mod. Phys.* **61**, 605–668 (1989).

[4] Casimir, H. B. G. Note on the conduction of heat in crystals. *Physica* **5**, 495–500 (1938).

[5] C., Z. R. & Pohl, R. O. Thermal conductivity and specific heat of noncrystalline solids. *Phys. Rev. B* **4**, 2029–2041 (1971).

[6] Pop, E., Varshney, V. & Roy, A. K. Thermal properties of graphene: Fundamentals and applications. *MRS Bulletin* **37**, 1273–1281 (2012).

[7] Seol, J. H. *et al.* Two-dimensional phonon transport in supported graphene. *Science* **9**, 213–216 (2010).

[8] Slack, G. A. Thermal Conductivity of Pure and Impure Silicon, Silicon Carbide, and Diamond. *J. Appl. Phys.* **35**, 3460–3466 (1964).

[9] Mazza, F., Bosisio, R, Benenti, G., Giovannetti, V., Fazio, R., & Teddei, F. Thermoelectric efficiency of three-terminal quantum thermal machines. *New J. Phys.* **16**, 085001 (2014).

[10] Beenakker, C. W. J. Quantum transport in semiconductor-superconductor microjunctions. *Phys. Rev. B* **46**, 12841–12844 (1992).

[11] Blonder, G. E., Tinkham, M. & Klapwijk, T. M. Transition from metallic to tunneling regimes in superconducting micro-constrictions' excess current, charge imbalance, and supercurrent conversion. *Phys. Rev. B* **25**, 4515–4532 (1982).

[12] Sun, Q.-F., Wang, J. & Lin, T.-H. Resonant Andreev reflection in a normal-metal-quantum-dot-superconductor system. *Phys. Rev. B* **59**, 3831–3840 (1999).

[13] Deacon, R. S. *et al.* Cooper pair splitting in parallel quantum dot Josephson junctions. *Nature Commun.* **6**, 7446 (2015).

[14] Gramich, J., Baumgartner, A. & Schönenberger, C. Andreev bound states probed in three-terminal quantum dots. *Phys. Rev. B* **96**, 195418 (2017).

[15] Pershoguba, S.S., & Glazman, L.I. Thermopower and thermal conductance of a superconducting quantum point contact. *Phys. Rev. B* **99**, 134514 (2019).

[16] Gramich, J., Baumgartner, A. & Schönenberger, C. Resonant and inelastic Andreev tunneling observed on a carbon nanotube quantum dot. *Phys. Rev. Lett.* **115**, 216801 (2015).

[17] Recher, P., Sukhorukov, E. V. & Loss, D. Andreev tunneling, Coulomb blockade, and resonant transport of nonlocal spin-entangled electrons. *Phys. Rev. B* **63**, 165314 (2001).

[18] Deutscher, G. & Feinberg, D. Coupling superconducting-ferromagnetic point contacts by Andreev reflections. *Appl. Phys. Lett.* **76**, 487–489 (2000).

[19] Golubev, D. S., Kalenkov, M. S. & Zaikin, A. D. Crossed Andreev reflection and charge imbalance in diffusive normal-superconducting-normal structures. *Phys. Rev. Lett.* **103**, 067006 (2009).

[20] Golubev, D. S. & Zaikin, A. D. Cross-correlated shot noise in three-terminal superconducting hybrid nanostructures. *Phys. Rev. B* **99**, 144504 (2019).

[21] Michalek, G., Urbaniak, M., Bulka, B.R., Domanski, T., & Wysokinski, K.I. Local and nonlocal thermopower in three-terminal nanostructures. *Phys. Rev. B* **93**, 235440 (2016).

[22] Guttman, G.D., Ben-Jacob, E., Bergmann, D.J. Thermoelectric properties of microstructures with four-probe versus two-probe setups. *Phys. Rev. B* **53**, 15856–15862 (1996).

[23] Kirsanov, N. S., Tan, Z. B., Golubev, D. S., Hakonen, P. J. & Lesovik, G. B. Heat switch and thermoelectric effects based on Cooper-pair splitting and elastic cotunneling. *Phys. Rev. B* **99**, 115127 (2019).

[24] Sadovskyy, I. A., Lesovik, G. B. & Vinokur, V. M. Unitary limit in crossed Andreev transport. *New J. Phys.* **17**, 103016 (2015).
